# The second-generation antipsychotic drug aripiprazole modulates the serotonergic system in pancreatic islets and induces beta cell dysfunction in female mice

**DOI:** 10.1007/s00125-021-05630-0

**Published:** 2021-12-21

**Authors:** Diana Grajales, Patricia Vázquez, Mónica Ruíz-Rosario, Eva Tudurí, Mercedes Mirasierra, Vítor Ferreira, Ana B. Hitos, Dora Koller, Pablo Zubiaur, Juan C. Cigudosa, Francisco Abad-Santos, Mario Vallejo, Iván Quesada, Boaz Tirosh, Gil Leibowitz, Ángela M. Valverde

**Affiliations:** 1 Instituto de Investigaciones Biomédicas Alberto Sols, Consejo Superior de Investigaciones Científicas (CSIC), Madrid, Spain; 2 CIBER de Diabetes y Enfermedades Metabólicas Asociadas (CIBERDEM), Instituto de Salud Carlos III, Madrid, Spain; 3 NIMGenetics, Madrid, Spain; 4 Instituto de Investigación, Desarrollo e Innovación en Biotecnología Sanitaria de Elche (IDiBE), Universidad Miguel Hernández, Elche, Spain; 5 Clinical Pharmacology Department, Hospital Universitario de La Princesa, Instituto de Investigación Sanitaria La Princesa, Madrid, Spain; 6 The Institute of Drug Research, The Hebrew University of Jerusalem, Jerusalem, Israel; 7 Endocrinology and Metabolism Service, Department of Medicine, Hadassah-Hebrew University Medical Center, Jerusalem, Israel

**Keywords:** Beta cell dysfunction, Beta cell mass, Insulin secretion, Islets, Schizophrenia, Second-generation antipsychotics, Type 2 diabetes

## Abstract

**Aims/hypothesis:**

Second-generation antipsychotic (SGA) drugs have been associated with the development of type 2 diabetes and the metabolic syndrome in patients with schizophrenia. In this study, we aimed to investigate the effects of two different SGA drugs, olanzapine and aripiprazole, on metabolic state and islet function and plasticity.

**Methods:**

We analysed the functional adaptation of beta cells in 12-week-old B6;129 female mice fed an olanzapine- or aripiprazole-supplemented diet (5.5–6.0 mg kg^−1^ day^−1^) for 6 months. Glucose and insulin tolerance tests, in vivo glucose-stimulated insulin secretion and indirect calorimetry were performed at the end of the study. The effects of SGAs on beta cell plasticity and islet serotonin levels were assessed by transcriptomic analysis and immunofluorescence. Insulin secretion was assessed by static incubations and Ca^2+^ fluxes by imaging techniques.

**Results:**

Treatment of female mice with olanzapine or aripiprazole for 6 months induced weight gain (*p*<0.01 and *p*<0.05, respectively), glucose intolerance (*p*<0.01) and impaired insulin secretion (*p*<0.05) vs mice fed a control chow diet. Aripiprazole, but not olanzapine, induced serotonin production in beta cells vs controls, likely by increasing tryptophan hydroxylase 1 (TPH1) expression, and inhibited Ca^2+^ flux. Of note, aripiprazole increased beta cell size (*p*<0.05) and mass (*p*<0.01) vs mice fed a control chow diet, along with activation of mechanistic target of rapamycin complex 1 (mTORC1)/S6 signalling, without preventing beta cell dysfunction.

**Conclusions/interpretation:**

Both SGAs induced weight gain and beta cell dysfunction, leading to glucose intolerance; however, aripiprazole had a more potent effect in terms of metabolic alterations, which was likely a result of its ability to modulate the serotonergic system. The deleterious metabolic effects of SGAs on islet function should be considered while treating patients as these drugs may increase the risk for development of the metabolic syndrome and diabetes.

**Graphical abstract:**

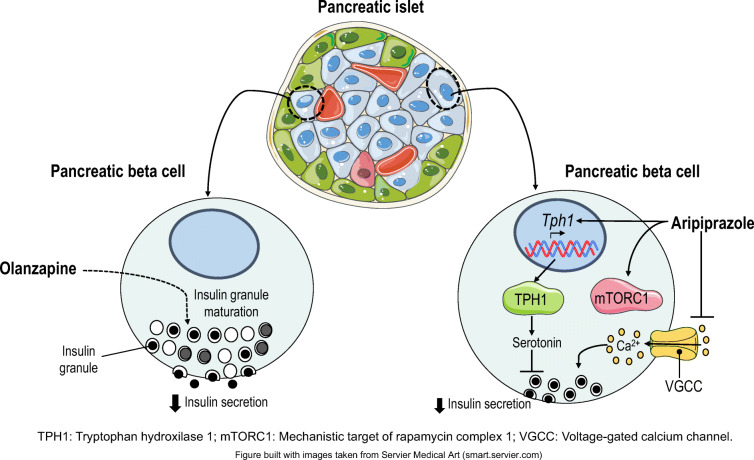

**Supplementary Information:**

The online version contains peer-reviewed but unedited supplementary material available at 10.1007/s00125-021-05630-0.

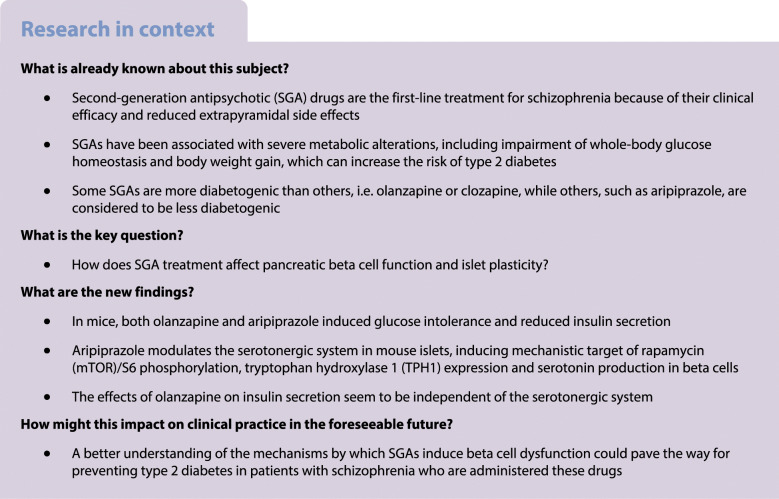



## Introduction

In recent years, an increased incidence of type 2 diabetes in patients taking chronic pharmacological treatment has been reported [1]. In patients receiving second-generation antipsychotic (SGA) drugs [2, 3], the first-line treatment for schizophrenia, the increase in incidence varies between 10% and 20%. SGAs induce metabolic alterations, including weight gain, hyperglycaemia, insulin resistance and dyslipidaemia, which increase the risk for cardiovascular disease [2]. In a large cohort of drug-naive individuals with schizophrenia, the incidence of type 2 diabetes was augmented in those prescribed the SGA olanzapine [4]. Rajkumar et al reported that the SGAs olanzapine and aripiprazole doubled the risk for developing type 2 diabetes, whereas the first in class antipsychotic, clozapine, increased the risk by fourfold [5]. Female individuals are more susceptible to the metabolic side effects of SGAs and, therefore, preclinical studies are often performed on female rodents [6].

SGAs act through a broad range of receptors, including dopamine D1– D4 receptors (D1R– D4R), serotonin receptors (5-hydroxytryptamine [5-HT])_1A_, 5-HT_2A_, 5-HT_2C_, 5-HT_3_, 5-HT_6_ and 5-HT_7_), histamine H_1_ receptor (H_1_R) or muscarinic M1–M5 receptors (M1R–M5R) [7]. Several studies have tested SGA drug-induced effects on whole-body glucose homeostasis [8]; however, their impact on beta cell function remains unclear [9]. Beta cells express different serotonergic receptors and synthesise, store and release serotonin in response to glucose, but the effects of SGAs on serotonin biosynthesis and signalling in islets and their impact on insulin secretion are not clear [10]. As reviewed previously [8], olanzapine has higher antagonistic activity against serotonin 5-HT_2A_ receptors and the dopamine receptor D2R, but is also antagonistic against D3R and D4R, 5-HT_3_ and 5-HT_6_ receptors, H_1_R, α1-adrenergic receptors and M1R–M5R. On the other hand, aripiprazole has partial agonistic activity for the dopamine receptors D2R, D3R and D4R, 5-HT_1A_ and 5-HT_2C_ receptors, and α1-adrenergic receptors, and also exhibits 5-HT_2A_ and 5-HT_7_ receptor antagonism.

Herein, we used the chemically unrelated SGAs, olanzapine (a commonly prescribed SGA that is highly diabetogenic) and aripiprazole (the metabolic side effects of which are less well-known) to study the effects of prolonged treatment with SGAs on blood glucose levels, islet morphometry and beta cell function in female mice.

## Methods

### Animals

Animal experiments were approved by the Animal Ethics Committees of the Spanish National Research Council and Comunidad de Madrid in accordance with Spanish (RD 53/2013) and European Union (63/2010/EU) legislation (PROEX 037/17).

Details of the B6;129 mice used in this study have been previously reported [11]. Mice were housed in a pathogen-free facility in temperature-, humidity- and light-controlled rooms (with a 12 h light–dark cycle), with free access to food and water. Ninety female mice, aged 12 weeks, were randomly allocated into three experimental groups; mice received a standard chow diet (SAFE A04; Scientific Diets [SAFE], France), or the same diet supplemented with olanzapine (GP8311; Glentham Life Sciences, UK) or aripiprazole (AC457990010; ACROS Organics, ThermoFisher Scientific, USA) (both 40 mg/kg chow diet). Dosage (5.5–6.0 mg kg^−1^ day^−1^) was calculated considering daily food intake. After 6 months on the diet, mice were euthanised by cervical dislocation and pancreatic islets, whole pancreases, white adipose tissue (WAT) depots (epididymal WAT [eWAT] and inguinal WAT [iWAT]) and blood were collected and processed for analysis. As a positive control for serotonin expression in islets we used 12-day pregnant B6 female mice, aged 16 weeks, bred in-house with B6 male mice.

### Analysis of olanzapine and aripiprazole in plasma

A simple and sensitive LC-MS/MS method (Agilent Technologies, Spain) was used for simultaneous determination of aripiprazole and olanzapine levels in plasma, as reported previously [12] and detailed in the electronic supplementary material (ESM) Methods.

### Food intake measurement

Food intake was measured manually using KERN PCB2500-2 scales (KERN, Germany) during the first month of treatment in mice housed in group cages and the mean food intake per mouse and per day was calculated.

### Metabolic assays

After 6 months on the diets, metabolic assays were performed, including i.p. GTT, i.p. ITT, glucose-stimulated insulin secretion (GSIS) and indirect calorimetry (see ESM Methods). In brief, for GTT and GSIS analysis, after 16 h of fasting, D-(+)-Glucose (2 g/kg body weight; G8270; Sigma-Aldrich, USA) was injected into mice and tail vein blood samples were collected at 0–120 min post-injection. For ITTs, after 4 h of fasting, human recombinant insulin (Actrapid; 0.75 U/kg body weight; Novo Nordisk, Denmark) was injected into mice and tail vein blood samples were collected at 0–90 min post-injection. Plasma glucose and insulin levels were measured via glucometer (Accu-Check Aviva; Roche Diagnostics, Switzerland) and ELISA (10-1247-01; Mercodia, Sweden), respectively. Indirect calorimetry analysis was carried out during light and dark cycles using the TSE Phenomaster monitoring system (TSE Systems, Germany). Oxygen consumption and CO_2_ release were measured, and respiratory exchange ratio (RER) was determined as $\dot{V}\mathrm{C}{\mathrm{O}}_2$/O_2_. Energy expenditure (EE) was calculated as EE = (3.185 + 1.232 × RER) × $\dot{V}{\mathrm{O}}_2$. Total locomotor activity was simultaneously measured using an infrared photocell beam interruption method, carried out using the TSE Phenomaster, as described previously [13]. Analysis was performed using the TSE Phenomaster Mouse software V5.1.7 (TSE Systems).

### Pancreatic islet isolation and culture

Islets were isolated by collagenase P (11215809103; Roche, Germany) digestion (13.5 U/ml in cold Hank’s buffer), as described previously [14]. For ex vivo experiments, islets were recovered overnight at 37°C and 5% CO_2_ in complete RPMI-1640 medium (2 mmol/l l-glutamine, 1 mmol/l sodium pyruvate, 50 μmol/l β-mercaptoethanol, 10 mmol/l HEPES and 10% [vol./vol.] FBS) containing 5.6 mmol/l glucose. The next day, islets were pooled, randomised and incubated with 6 μmol/l olanzapine or aripiprazole (dissolved in DMSO; D8418; Sigma-Aldrich), 1–500 μmol/l serotonin (14927; Sigma-Aldrich) (1–24 h incubation) or 10 μmol/l 4-Chloro-dl-phenylalanine (PCPA; C6506; Sigma-Aldrich). Control islets were treated with 0.01% (vol./vol.) DMSO.

### Static incubations

For each individual mouse, 3–6 groups of three islets matched by size were placed in each well of a 96-well plate. Islets were pre-incubated for 1 h at 37°C and 5% CO_2_ in KRB containing 2.8 mmol/l glucose, 115 mmol/l NaCl, 5 mmol/l KCl, 1.2 mmol/l NaHCO_3_, 1.1 mmol/l MgCl_2_, 1.2 mmol/l NaH_2_CO_4_, 2.5 mmol/l CaCl_2_, 25 mmol/l HEPES and 0.25% (wt/vol.) BSA. Incubations were then performed using 2.8 mmol/l or 16.7 mmol/l glucose at 37°C, 5% CO_2_ for 1 h. Insulin levels were determined by ELISA (Mercodia) and values were normalised to islet number.

### Insulin content

Insulin was extracted from 20 islets/mice using glycine/NP-40 lysis buffer (200 mmol/l glycine, 0.5% NP-40; pH 8.8) and measured by ELISA.

### Intracellular Ca^2+^ imaging

Islets treated ex vivo with SGAs were pre-incubated with Fura-2 AM (F11212; ThermoFisher Scientific) and perfused with KRB containing glucose (2.8 mmol/l or 16.7 mmol/l). Fluorescence measurements were obtained at excitation wavelengths of 340 nm and 380 nm with an Axiovert 200 inverted microscope (Zeiss, Germany) with appropriate filters. Data acquisition was performed with the Aquacosmos 2.6 software (Hamamatsu Photonics, Japan). Recordings were expressed as the ratio of fluorescence at 340 nm and 380 nm (F340/380).

### Immunohistochemistry

Pancreases were fixed in Bouin’s solution (HT10132; Sigma-Aldrich) overnight at 4°C. Paraffin embedding and tissue sectioning were performed as described previously [15]. Longitudinal pancreatic sections of 6 μm thickness, generated every 80 μm, were hydrated and pre-treated by boiling for 20 min in a microwave in antigen-retrieval solution containing 100 mmol/l sodium citrate dehydrate (pH 6; W302600; Sigma-Aldrich) supplemented with 0.05% (vol./vol.) Tween-20. Insulin and glucagon expression was analysed by immunohistochemistry staining using primary antibodies against insulin and glucagon, and secondary biotinylated antibodies diluted in PBS (ESM Table 1). Pancreatic sections were then processed for diaminobenzidine (DAB)-immunoperoxidase staining (SK-4100; Vector Laboratories, USA) and counterstained with Mayer’s Hematoxylin (H3136; Sigma-Aldrich). Images were examined using a Axiophot Zeiss light microscope and captured with a DP70 digital camera (Olympus, Japan). Insulin and glucagon staining and total pancreatic area were quantified by ImageJ software version 1.52a (NIH, USA). Morphometric analysis of the pancreas is described further in ESM Methods.

### Immunofluorescence of pancreatic sections and islets

Pancreatic sections were processed as described above using antibodies against insulin, glucagon, serotonin, p-S6 and Ki67, and secondary Alexa-Fluor conjugated antibodies (see ESM Methods for further details). For in toto islet immunostaining, 20 islets were handpicked, placed in μ-Slide 8-well plates (80826; Ibidi, Germany) and processed for insulin and serotonin immunostaining, as detailed in ESM Methods. Antibody details are listed in ESM Table 1. Immunofluorescence was examined using an epifluorescence microscope (Nikon 90i; Olympus) and images were taken with a digital camera (Nikon DS-2Mv, Japan). The percentage of beta cells co-expressing insulin and Ki67, p-S6 or serotonin was obtained by dividing the number of positive cells for each staining by the total number of insulin-positive cells in each islet.

### Ultrastructural analysis by transmission electron microscopy

For transmission electron microscopy (TEM) analysis, pools of 300 pancreatic islets from three mice per condition were processed as described in ESM Methods. Tissue sections were examined using a Zeiss Libra 120 transmission electron microscope and TEM images were taken with an electron multiplying charge coupled device (EMCCD) camera (Albert Tröndle, Germany). The number and type of the secretory granules in beta cells (*n* = 10 beta cells from three independent mice/group) were assessed using ImageJ software (NIH). Insulin granules from beta cells were classified into four categories: mature (with an electron-dense core); immature (with a less electron-dense core); empty (lacking the core); and atypical (insulin granules with an irregular shape).

### Serotonin measurement

Supernatants collected from static incubation experiments were used for the measurement of serotonin levels using the ELISA Fast Track kit (BA E-8900; LDN, Germany). Values were normalised to islet number.

### Transcriptomic analysis of islets from treated mice by RNA-sequencing

Islets were isolated in TRIzol (15596026; ThermoFisher Scientific) and total RNA was extracted using the PureLink RNA Mini Kit (Invitrogen, USA). Total RNA expression was analysed using Illumina TruSeq Stranded RNASeq technology (Illumina, USA). The libraries were sequenced (2 × 100 bp) with a mean output of 40 million reads in a NovaSeq 6000 sequencer (Illumina). After a quality control check with FastQC (www.bioinformatics.babraham.ac.uk/projects/fastqc, access date 27 May 2019), the reads were aligned to reference transcripts with the Kallisto algorithm [16], which provides a matrix of estimated counts per transcript as the output. Exploratory analyses included principal component analysis (PCA) and hierarchical clustering (HC). Transcriptomic analyses were performed with the DESeq2 package [17], for which differentially expressed genes (DEGs) were described as those with an adjusted *p* value (*p*-adj) of <0.1 when performing a Wald test between two conditions and a Benjamini–Hochberg adjustment. Over-representation analyses (ORAs) of the DEGs were completed with the WEB-based GEne SeT AnaLysis Toolkit (WebGestalt) [18].

### Western blotting

Protein levels were assessed in pancreatic islets using antibodies against IRS-2, mechanistic target of rapamycin (mTOR), p-mTOR (Ser2448), S6K1, p-S6K1 (Thr389), p-S6 ribosomal protein, tryptophan hydroxylase 1 (TPH1) and vinculin (ESM Table 1). Immunoreactivity was detected by chemiluminescence, using Clarity Western ECL Substrate (1705061; Bio-Rad, Germany). Densitometric analysis of the bands was performed using ImageJ software (NIH). The protocol is fully described in ESM Methods.

### Quantitative real-time PCR

Gene expression was determined by quantitative real-tim**e** PCR (RT-qPCR) using Power SYBR Green PCR Master Mix (4367659; ThermoFisher Scientific) and 7900HT Fast Real-Time PCR System (ThermoFisher Scientific), as described in ESM Methods. Primer sequences are shown in ESM Table 2.

### Statistical analysis

Statistical analysis was performed using Prism 8 (Graph software, USA). Datasets were first analysed for normal distribution. For data with parametric distributions, unpaired Student’s *t* test was used to compare mean differences between two groups, and for three or more groups, one-way ANOVA with Bonferroni post hoc test was used. For data with non-parametric distributions, differences between groups were examined with Mann–Whitney *U* test for two groups, or Kruskal–Wallis test for three or more groups. Two-way ANOVA was employed to compare two different categorical, independent variables. Where other statistical analyses have been used, this has been indicated in the figure legends. Data are expressed as mean ± SEM. Tests were two-sided and *p*<0.05 was considered statistically significant. Mice and islets were randomly and blindly distributed for the treatments by experimenters. Experimenters were not blind in outcome assessment.

## Results

### Alterations in body weight, adiposity, energy balance and glucose metabolism in female mice fed an antipsychotic drug-supplemented diet

**Fig. 1:**
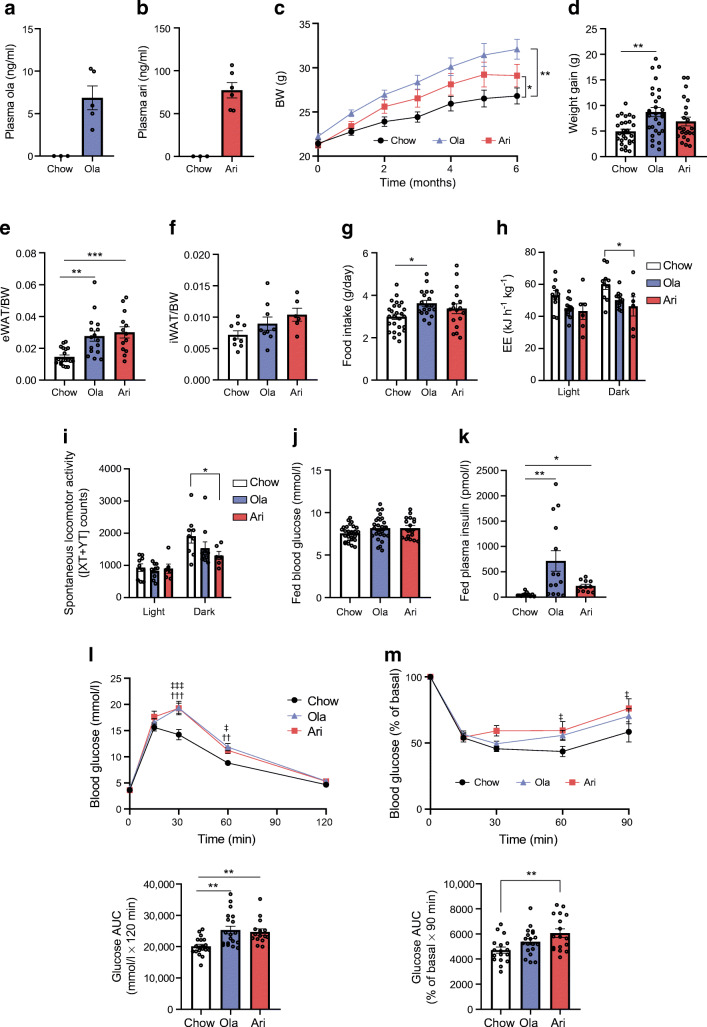
Effects of olanzapine (ola)- and aripiprazole (ari)-supplemented diet on body weight (BW), adiposity, energy balance and glucose metabolism in female mice*.* (**a**, **b**) Plasma levels of ola (**a**) and ari (**b**) in mice after 6 months of treatment with antipsychotic drug-supplemented diets (*n*=3–6 mice/group). (**c**) BW monitored monthly and (**d**) BW gain in the last month of the treatment in mice fed an ari- or ola-supplemented diet (*n*=28 control mice, *n*=29 ola-treated mice, *n*=23 ari-treated mice) (**e**) Epididymal WAT (eWAT) and (**f**) iWAT normalised to BW (*n*=6–19 mice/group). (**g**) Food intake during the first month of treatment (*n*=17–26 mice/group). (**h**) EE and (**i**) locomotor activity (presented as [XY+YT] counts, indicating the total number of times mice cross the infrared sensors that border the measuring cage on the X and Y planes) measured at the end of the treatment period by indirect calorimetry (*n*=6–13 mice/group). Light cycle: 08:00–20:00 hours; dark cycle: 20:00–08:00 hours. (**j**) Fed blood glucose (mmol/l) and (**k**) fed plasma insulin (pmol/l) levels (*n*=11–27 mice/group). (**l**) i.p. GTT and the respective AUC (*n*=15–19 mice/group). The AUC was calculated from 0 to 120 min, according to the trapezoidal rule. (**m**) i.p. ITT and the respective AUC (*n*=17–19 mice/group). The AUC was calculated from 0 to 90 min, according to the trapezoidal rule. All data are presented as mean±SEM. *p* values were determined by one-way (**d**, **e**, **f**, **g**, **j**, **k**, **l** (lower), **m** (lower)) or two-way (**c**, **h**, **i**, **l** (upper), **m** (upper)) ANOVA and Bonferroni post hoc test. *p<0.05, **p<0.01, ***p<0.001 vs mice fed a chow diet; ^††^*p*<0.01, ^†††^*p*<0.001, ola-treated mice vs mice fed a chow diet; ^‡^*p*<0.05, ^‡‡‡^*p*<0.001, ari-treated mice vs mice fed a chow diet

Female mice were fed an olanzapine- or aripiprazole-supplemented diet (40 mg/kg) for 6 months. Figure 1a,b shows plasma drug levels at the end of the treatment. Olanzapine-treated mice gained 8.70 ± 0.88 g of body weight compared with a 4.90 ± 0.47 g gain in controls fed a chow diet (*p*<0.01). Aripiprazole-treated mice also gained more weight than the controls over the treatment period (*p*<0.05) but, as body weight stabilised in the last month of the treatment in this group, there was less body weight gain compared with olanzapine-treated mice (*p*>0.05) (Fig. 1c,d). Both olanzapine- (*p*<0.01) and aripiprazole-treated (*p*<0.001) mice had a significant increase in visceral adiposity and showed a slight, but not significant, increase in iWAT/body weight ratio vs controls (Fig. 1e,f).

We further studied the effects of the two SGAs on food intake. Consistent with a previous report [19], food consumption was higher in the olanzapine-treated group compared with the control group (*p*<0.05; Fig. 1g), whereas no differences were found between aripiprazole-treated mice vs control or olanzapine-treated groups. EE and spontaneous locomotor activity were lower in the dark phase in olanzapine- and aripiprazole-treated mice vs control mice, although this difference was only statistically significant for the aripiprazole-treated group vs controls (*p*<0.05; Fig. 1h,i).

Fed and fasting blood glucose levels did not differ between groups (Fig. 1j, ESM Fig. 1b). However, fed plasma insulin levels were higher in mice receiving the olanzapine- (*p*<0.01) or aripiprazole-supplemented diet (*p*<0.05; Fig. 1k), whereas fasting insulin was similar between groups (ESM Fig. 1c), suggesting increased insulin resistance or impairment of insulin clearance with SGA treatment. We further assessed the effects of SGAs on glucose tolerance and insulin sensitivity. The GTT showed that olanzapine- and aripiprazole-fed mice developed glucose intolerance (Fig. 1l). The ITT revealed that, although insulin sensitivity was reduced in both groups of treated mice, only the difference between the aripiprazole-treated and control groups was statistically significant (*p*<0.01; Fig. 1m). Collectively, these findings suggest that both SGAs induce alterations in glucose homeostasis, despite the fact that aripirazole treatment was associated with less weight gain.

### Olanzapine and aripiprazole impaired beta cell function and altered islet morphology in female mice

**Fig. 2:**
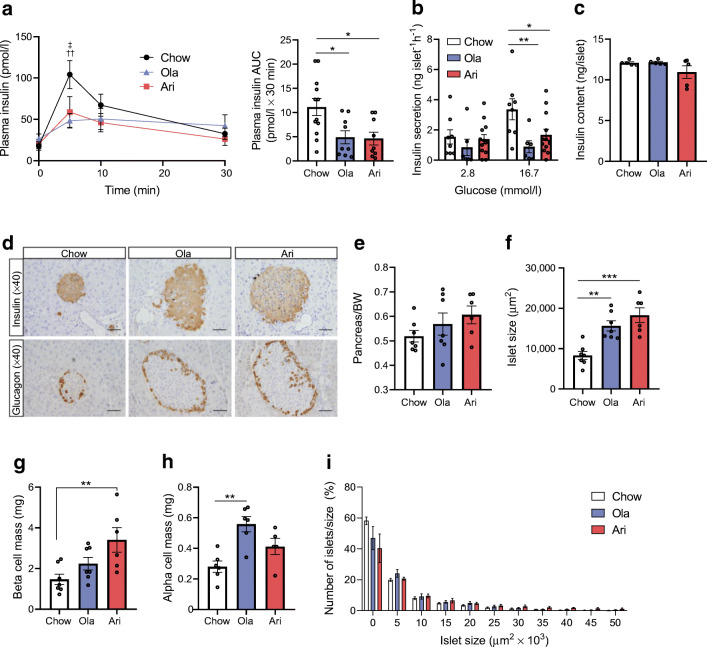
Effects of olanzapine (ola)- or aripiprazole (ari)-supplemented diet on GSIS and islet morphology in female mice. (**a**) In vivo GSIS and the respective AUC, calculated from 0 to 30 min values, according to the trapezoidal rule (*n*=9–12 mice/group). (**b**) Ex vivo GSIS, performed using 3–6 technical replicates for each condition and mouse (*n*=6–13 mice/group). Insulin secretion was corrected for islet number. (**c**) Insulin content in islets. Twenty islets per mouse were lysed and the insulin content was normalised to islet number (*n*=5 mice/condition). (**d**) Representative images of pancreatic islets stained with insulin and glucagon; scale bars, 50 μm; magnification ×40. (**e**) Pancreas weight normalised to body weight (BW). (**f**) Islet size (μm^2^). (**g**) Beta cell mass (mg) (*n*=7 control, *n*=7 ola-treated mice, *n=*6 ari-treated mice) and (**h**) alpha cell mass (mg) (*n*=6 control, *n*=6 ola-treated mice, *n=*5 ari-treated mice). (**i**) Islet size distribution (*n*=7 control, *n*=7 ola-treated mice, *n*=6 ari-treated mice). Between 8 and 12 pancreatic sections per mouse, generated every 80 μm, were analysed. At least 300 islets per mice were counted for determination of islet size. The differences between the distribution of islet size in ola- and ari-treated mouse samples were significant vs the distribution of islet size in samples from mice fed a chow diet (*p*<0.001, by χ^2^ test). All data are presented as mean±SEM. *p<0.05, **p<0.01, ***p<0.001 vs mice fed a chow diet, by one-way ANOVA and Bonferroni post hoc test for the AUC graph in (**a**, right**)** and in (**c**, **e**, **f**, **g**, **h**) or by two-way ANOVA in (**a**, left, **b**); ^††^*p*<0.01, ola vs chow; ^‡^*p*<0.05, ari vs chow

Olanzapine and aripiprazole treatment markedly impaired GSIS in vivo (AUC *p*<0.05; Fig. 2a), indicating impaired beta cell function. Consistently, ex vivo static incubations showed that GSIS was inhibited in islets of both olanzapine- (*p*<0.01) and aripiprazole-treated animals (*p*<0.05), as compared with islets from chow-diet-fed mice (Fig. 2b), without affecting islet insulin content (Fig. 2c).

To determine the mechanism underlying beta cell dysfunction in response to SGA treatment, islet morphometry was analysed (Fig. 2d–i). Islet size markedly increased in mice treated with olanzapine (*p*<0.01) or aripiprazole (*p*<0.001) vs controls (Fig. 2f). Beta cell mass was increased by twofold in aripiprazole-treated mice compared with chow-fed mice (*p*<0.01), but this effect was not observed in olanzapine-treated mice (Fig. 2g). Interestingly, alpha cell mass was twofold higher in olanzapine-treated mice than in the controls (Fig. 2h), which was associated with a non-significant increase in alpha cell area without changes in islet cell composition (ESM Fig. 2). Comparative analysis of islet size distribution among groups confirmed the increased number of larger sized islets in mice treated with SGAs vs controls (*p*<0.001, analysed by χ^2^ test; Fig. 2i). Ultrastructural TEM analysis showed smaller numbers of mature insulin granules (*p*=0.09) and more empty granules (*p*=0.09) in beta cells from olanzapine-treated mice vs controls (ESM Fig. 2f).

### Effects of antipsychotic drug-supplemented diet on beta cell proliferation and size in female mice

**Fig. 3:**
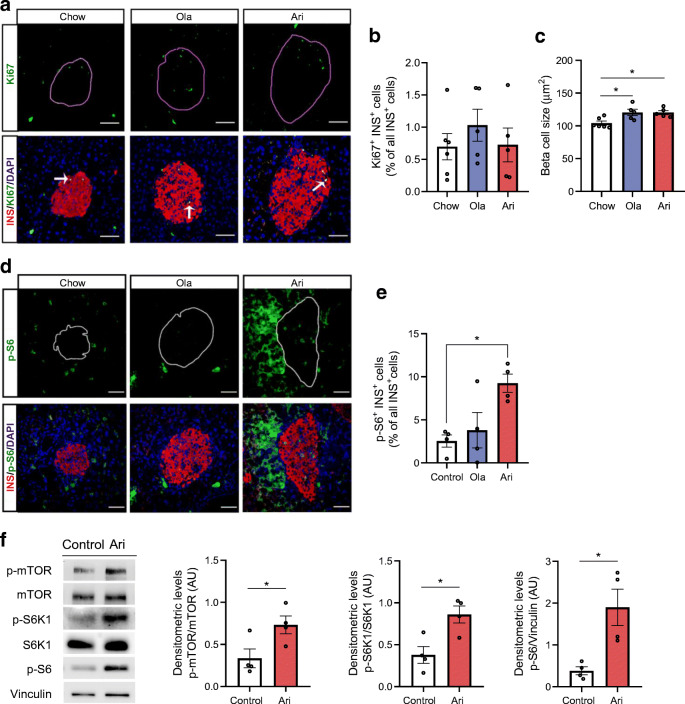
Analysis of beta cell proliferation and size and phosphorylation of S6 ribosomal protein in islets from female mice after 6 months of treatment with an antipsychotic drug-supplemented diet. (**a**) Confocal images of Ki67 (green) immunofluorescence. Ki67 co-localisation with insulin (INS)^+^ cells (red) is indicated (white arrows); scale bars, 50 μm; magnification ×40. (**b**) Percentage of Ki67^+^INS^+^ cells and (**c**) beta cell size (μm^2^). A total of 43.85 ± 4.83 islets were analysed for Ki67 expression and beta cell size (*n*=5–6 mice/group). (**d**) Confocal microscopy images of islets double immunostained with antibodies against p-S6 (green) and INS (red); scale bars, 50 μm; magnification ×40. (**e**) Percentage of p-S6^+^INS^+^ cells. All islets within two pancreatic sections per mouse (*n*=4 mice/group) were analysed, with each section being generated every 200 μm. A total of 22.83 ± 1.98 islets were quantified for p-S6 staining. (**f**) Islets were incubated with 6 μmol/l aripiprazole (ari) or vehicle (0.01% DMSO [control]) for 16 h and phosphorylation levels of mTOR, S6K1 and S6 were analysed by western blot. Representative blots of mTOR, S6K1 and S6 phosphorylation levels in independent pools of 200–300 islets isolated from *n*=4–6 mice are shown. Quantification of protein levels is also shown (*n*=4 independent experiments). Data are presented as mean±SEM. AU, arbitrary units; ola, olanzapine. *p<0.05 vs mice fed a chow diet, analysed by one-way ANOVA test and Bonferroni post hoc test in (**b**, **c**), by Kruskal–Wallis test and Bonferroni post hoc test in (**e**), or unpaired Student’s *t* test in (**f**)

Consistent with a report in adult animals [20], beta cell proliferation, assessed by Ki67 immunostaining, was low and no differences were found between groups (Fig. 3a,b). Notably, beta cell size was increased in both olanzapine- and aripiprazole-treated mice (*p*<0.05; Fig. 3c). mTOR complex 1 (mTORC1), a key regulator of cell size, plays an important role in beta cell compensation under stress conditions [21]. Immunostaining with an antibody against phosphorylated ribosomal protein S6, a downstream mTORC1 target, showed increased mTORC1 activity in beta cells from aripiprazole-treated mice (*p*<0.05), but not from mice receiving olanzapine (Fig. 3d,e). Thus, mTORC1 might have a role in mediating beta cell compensation in aripiprazole-treated mice. Islets were then treated ex vivo with aripiprazole for 16 h and mTORC1 activity was assessed by western blotting for mTOR, S6K1 and S6 phosphorylation. Treatment with aripiprazole increased mTOR/S6K1/S6 phosphorylation (Fig. 3f), indicating stimulation of the mTORC1 signalling pathway.

### Ex vivo treatment of pancreatic islets with olanzapine and aripiprazole impairs GSIS

**Fig. 4:**
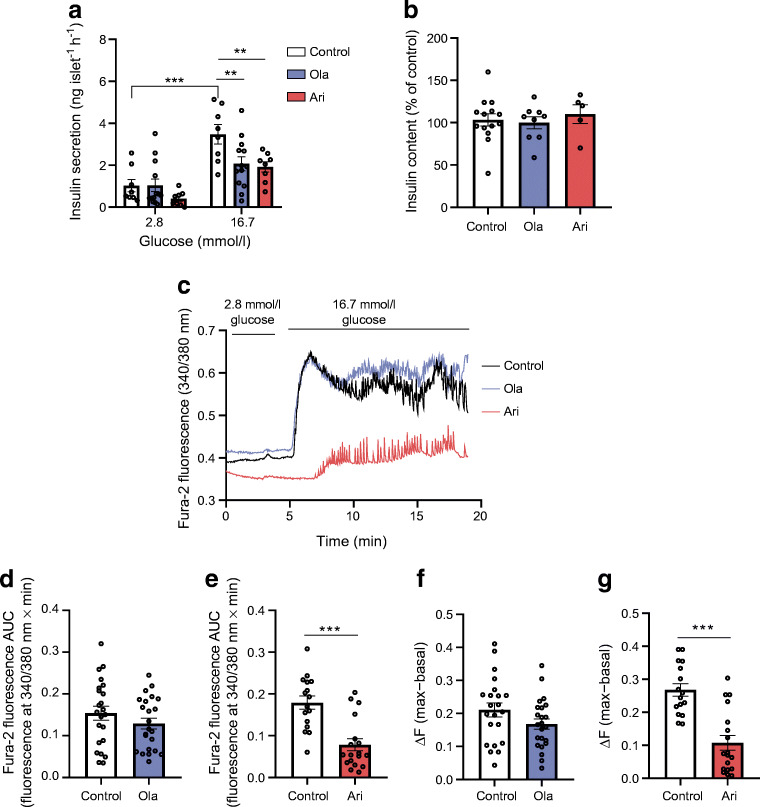
Effect of ex vivo treatment of pancreatic islets with olanzapine (ola) or aripiprazole (ari) on insulin secretion and Ca^2+^ signalling*.* (**a**) GSIS analysis performed in islets treated with either ola or ari (6 μmol/l) or vehicle (0.01% DMSO [control]) for 24 h. We performed 3–6 technical replicates for each condition and mouse (*n*=8–13 mice/group). (**b**) Insulin content in islets. Twenty islets per condition were lysed and the insulin content was measured and normalised to insulin content in control islets. Statistically significant differences were not observed between groups, as determined by one-way ANOVA (*n*=5–14 mice/group). (**c**) Representative recordings of Fura-2 Ca^2+^ fluorescence in beta cells from islets in response to high glucose concentrations (16.7 mmol/l) under treatment with vehicle (0.01% DMSO [control]), 6 μmol/l ola or 6 μmol/l ari. (**d**, **e**) Quantification of the AUC of Fura-2 Ca^2+^ fluorescence per min in vehicle-treated islets vs islets treated with ola (**d**) or ari (**e**). (**f**, **g**) ΔF (maximum [max] – F_basal_) in control islets vs islets treated with ola (**f**) or ari (**g**). In (**d**–**g**) a total of 16–25 islets from *n*=6 mice were used in each condition. Data are presented as mean±SEM. **p<0.01, ***p<0.001 vs control, by two-way ANOVA and Bonferroni post hoc test in (**a**), Student’s *t* test in (**d**, **f**, **g**) or Mann–Whitney *U* test in (**e**)

Ex vivo GSIS analyses showed that both olanzapine and aripiprazole used at 6 μmol/l reduced insulin secretion in islets without affecting insulin content (Fig. 4a,b), suggesting direct inhibitory effects of these drugs on insulin secretion.

We next analysed Ca^2+^ signalling in islets exposed ex vivo to olanzapine or aripiprazole for 24 h. Islets treated with olanzapine showed a similar pattern of Ca^2+^ oscillations compared with control islets and no differences were found in the AUC/min, change in fluorescence (ΔF), basal fluorescence (F_basal_) or response time to high glucose (time islets take to respond to change in glucose concentration by opening voltage-gated Ca^2+^ channels) vs controls (Fig. 4c,d,f, ESM Fig. 3a,c). By contrast, aripiprazole-treated islets exhibited attenuated Ca^2+^ entry, as reflected by decreased ΔF and AUC/min, and delayed response to high glucose vs controls, while F_basal_ was similar between groups (Fig. 4e,g, ESM Fig. 3b,d). These data suggest that aripiprazole interferes with Ca^2+^ signalling in beta cells.

### Transcriptomic analysis in pancreatic islets from female mice fed an olanzapine- or aripiprazole-supplemented diet

**Fig. 5:**
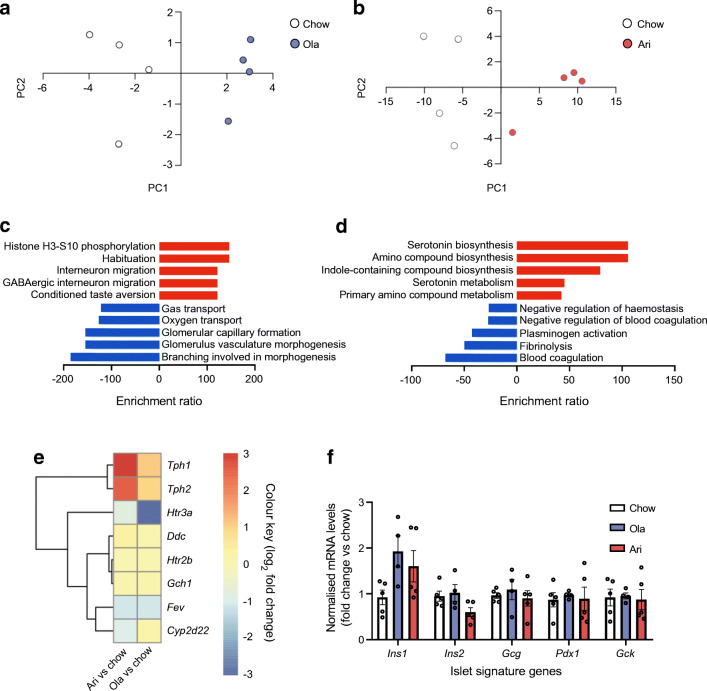
Transcriptomic analysis in pancreatic islets from female mice fed an olanzapine (ola)- or aripiprazole (ari)-supplemented diet. (**a**, **b**) PCA of samples from mice treated with ola (**a**) or ari (**b**) vs chow-fed mice. Each sample contained a pool of 500 islets from *n*=4 mice (*n*=4 samples/group). (**c**, **d**) ORA of ola-associated (**c**) and ari-associated (**d**) DEGs. (**e**) Heatmap of serotonin biosynthetic processes The log_2_ fold change in gene expression in islets treated with ari or ola vs islets from mice fed a chow diet is shown, with downregulated genes shown in blue and upregulated genes shown in red. (**f**) Analysis of common islet-related genes by RT-qPCR. Expression was normalised to the mean C_t_ values of two housekeeping genes (*Actb* and *Gapdh*). Each sample contained a pool of 500 islets from *n*=4 mice (*n*=4–5 samples/group). Data are presented as mean±SEM. Statistical significance differences were not observed in (**f**), as analysed by the Kruskal–Wallis test, or one-way ANOVA and Bonferroni post hoc test. GABA, γ-aminobutyric acid; PC, principal component

To identify the transcriptomic profile of mouse islets from SGA-treated mice we conducted RNA-sequencing (RNA-seq). PCA showed differential gene expression in islets from mice under SGA treatment (Fig. 5a,b). DEGs were identified by DESeq2 and classified as genes with *p*-adj<0.1 as assessed using a Wald test between two conditions with Benjamini–Hochberg adjustment. Fifteen genes were differentially expressed in islets from olanzapine-treated mice and 244 genes were dysregulated in islets from aripiprazole-treated mice (ESM Table 3, ESM Table 4). Islets from mice receiving a chow diet were used to identify baseline gene levels.

We conducted ORA to address the specific genetic signatures associated with olanzapine or aripiprazole treatment. However, because 15 DEGs (in the case of olanzapine) is a small number of genes for ORA, the analysis was performed with all genes with a fold change ≤ −1.5 or ≥1.5 and a *p* value <0.05, as assessed by the Wald test between two conditions, and relevant findings were further validated by RT-qPCR. A total of 289 genes dysregulated by aripiprazole and 136 by olanzapine were included in the ORA (data not shown). We found that the top-five upregulated pathways in the aripiprazole arm appeared to be related to serotonin biosynthesis (Fig. 5d). The heatmap of serotonin biosynthetic processes (gene set accession no.: GO:0042427, http://amigo.geneontology.org/amigo/term/GO:0042427/?q=DDC; access date 22 July 2019) shows that genes encoding the serotonin-synthetising enzymes *Tph1* and *Tph2* were upregulated in islets from aripiprazole-treated mice, whereas the gene encoding the *Htr3a* receptor was downregulated in islets of olanzapine-treated mice (Fig. 5e). Notably, transcriptional profiling showed no significant alterations in other genes related to islet function with SGA treatment (ESM Table 3, ESM Table 4). Figure 5f shows the RT-qPCR analysis of common islet genes.

### Effects of olanzapine and aripiprazole treatment on the expression of serotonin-related genes and serotonin levels in islets

**Fig. 6:**
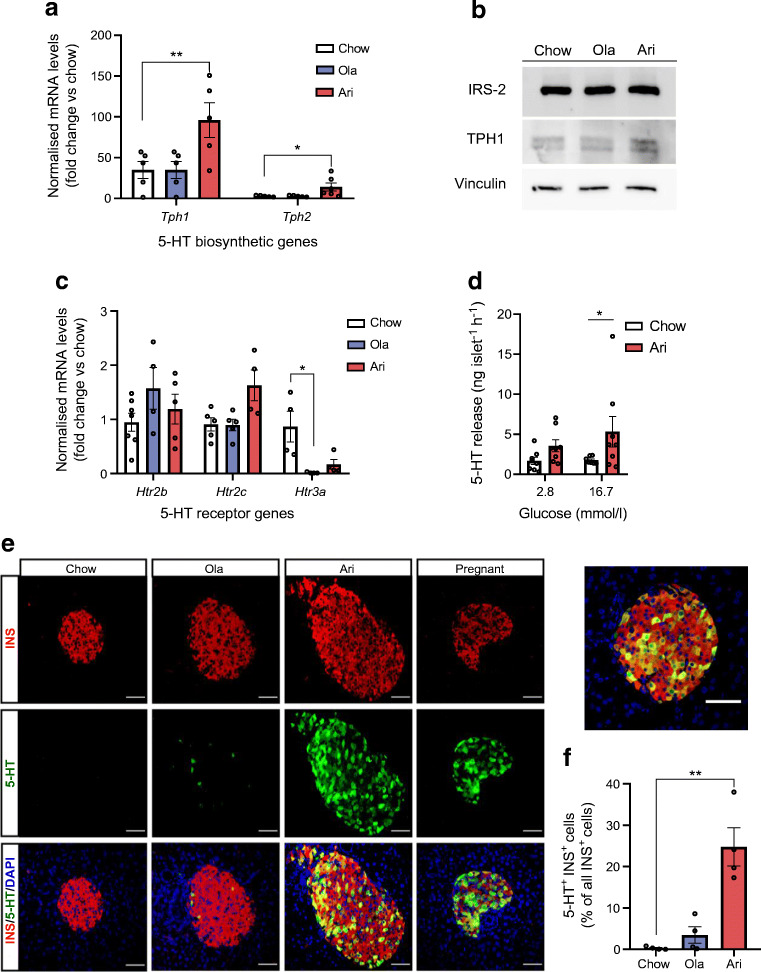
Effect of olanzapine (ola) or aripiprazole (ari) treatment on the expression of serotonergic system-related genes and serotonin (5-HT) levels in islets from female mice after 6 months of treatment with antipsychotic drug-supplemented diets. (**a**) RT-qPCR analysis of 5-HT-related biosynthetic gene expression normalised against median C_t_ values of two housekeeping genes (*Actb* and *Gapdh*). Each sample contained a pool of 500 islets from *n*=4 mice (*Tph1* analysis: *n*=5 samples/group; *Tph2* analysis: *n*=5 samples for control, *n*=5 samples for ola-treated mice and *n*=6 samples for ari-treated mice). (**b**) TPH1 protein expression by western blot. Each sample contained a pool of 500 islets from *n*=3 mice/group (control mice, or mice treated with ola or ari for 6 months). Vinculin and IRS-2 were used as loading controls. (**c**) mRNA expression of 5-HT receptor genes, analysed by RT-qPCR. Each sample contained a pool of 500 islets from *n*=4 mice (*n*=4–7 samples/group). (**d**) 5-HT release was measured by ELISA in the culture medium of islets previously challenged with 16.7 mmol/l glucose (*n*=8 mice/group). (**e**) Representative images of pancreatic islets expressing insulin (INS; red) and 5-HT (green), captured with confocal microscopy; scale bars, 50 μm; magnification ×40. A higher magnification of an islet co-expressing INS and 5-HT after ari treatment is also shown; scale bar, 50 μm; magnification ×40. (**f**) Percentage of 5-HT^+^INS^+^ cells. All islets within two pancreatic sections per mouse (*n*=4 mice/group) were analysed, with each section being generated every 200 μm. A total of 26.25 ± 3.28 islets were quantified for 5-HT expression. Data are presented as mean±SEM. *p<0.05, **p<0.01 vs mice fed a chow diet, analysed by one-way ANOVA and Bonferroni post hoc test in (**a**, **c**), two-way ANOVA and Bonferroni post hoc test in (**d**) or Kruskal–Wallis test (**f**)

In agreement with RNA-seq data, RT-qPCR showed that aripiprazole increased *Tph1* and *Tph2* expression vs controls (*p*<0.01 and *p*<0.05, respectively; Fig. 6a), along with an apparent increase in TPH1 protein levels (Fig. 6b). In addition, the expression of the serotonin receptor *Htr3a* was reduced in the olanzapine-treated group vs controls (*p*<0.05; Fig. 6c).

Serotonin has been implicated in beta cell compensation during pregnancy [22, 23]. Ex vivo experiments showed higher serotonin secretion in islets from aripiprazole-treated mice, both with 2.8 mmol/l and 16.7 mmol/l glucose treatment, with findings being significant following exposure to 16.7 mmol/l glucose (*p*<0.05; Fig. 6d). Immunofluorescence images confirmed higher serotonin levels in islets from mice that received aripiprazole compared with control mice (*p*<0.01; Fig. 6e,f). In fact, we observed that serotonin levels in islets from aripiprazole-treated mice appeared to be comparable with islets of pregnant mice (Fig. 6e). Overall, our findings indicate that aripiprazole treatment increased serotonin synthesis and secretion in islets.

### Aripiprazole increases serotonin generation and induces TPH1 expression in pancreatic islets

**Fig. 7:**
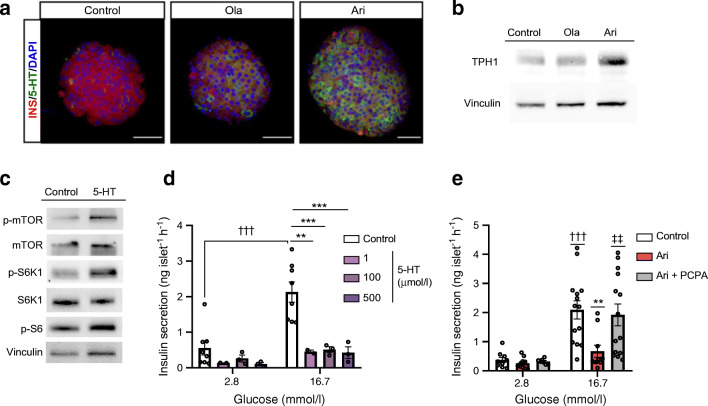
Effect of ex vivo treatment of pancreatic islets with olanzapine (ola) or aripiprazole (ari) on serotonin (5-HT) expression. (**a**) In toto islets were immunostained for insulin (INS; red) and 5-HT (green) after ex vivo treatment with ola or ari for 24 h. Images were captured by confocal microscopy; scale bar, 50 μm; magnification ×40. (**b**) TPH1 protein expression analysed by western blot (using vinculin as a loading control) after ex vivo treatment of islets with ola or ari for 24 h. Each sample (control [untreated islets], or islets treated ex vivo with ola or ari) contained a pool of 500 islets from *n*=12 mice. (**c**) Islets were incubated with 100 μmol/l 5-HT for 1 h and phosphorylation levels of mTOR, S6K1 and S6 were analysed by western blot. Each sample (control [untreated islets] or islets treated ex vivo with 5-HT) contained a pool of 200 islets from *n*=6 mice. (**d**) Ex vivo GSIS in pancreatic islets after 24 h of treatment with 5-HT. We performed 3–6 technical replicates for each condition and mouse (*n*=2–3 mice for serotonin, *n*=8 mice for control). (**e**) Ex vivo GSIS in pancreatic islets after 24 h of treatment of ari (6 μmol/l) and PCPA (10 μmol/l). Experiments were performed with at least 3–6 technical replicates per condition in pools of islets from *n*=10–14 mice (*n*=3 independent experiments). **p<0.01, ***p<0.001 vs control islets stimulated with 16.7 mmol/l glucose; ^†††^*p*<0.001 vs control islets stimulated with 2.8 mmol/l glucose; ^‡‡^*p*<0.01 vs islets treated with ari and stimulated with 16.7 mmol/l glucose; analysed by two-way ANOVA and Bonferroni post hoc test

In light of the in vivo findings showing that aripiprazole treatment increased *Tph1* mRNA (*p*<0.01) and that there was an apparent increase in THP1 protein levels, along with increased serotonin secretion (*p*<0.05) vs controls (Fig. 6a,b,d–f), we studied its direct effects on the serotonergic system in isolated islets. As shown in Fig. 7a,b, treatment with aripiprazole for 24 h appeared to increase serotonin and TPH1 protein levels vs controls.

Finally, we studied whether serotonin mediates the effects of aripiprazole on mTORC1 activity and beta cell function. Our findings suggest that treatment with serotonin (100 μmol/l) for 1 h increased mTORC1 activity, as reflected by apparent increases in mTOR, S6K1 and S6 phosphorylation vs controls (Fig. 7c). Moreover, treatment of islets with serotonin for 24 h inhibited GSIS vs controls (Fig. 7d), as previously reported [24, 25]. Importantly, co-treatment with aripiprazole and the TPH1 inhibitor PCPA prevented the negative effect of aripiprazole on insulin secretion (Fig. 7e). Collectively, we suggest that aripiprazole increases serotonin biosynthesis and secretion in islets and mediates mTORC1 activation and, probably, beta cell hypertrophy, while impairing insulin secretion.

## Discussion

This study provides novel findings on the effect of the SGAs olanzapine and aripiprazole in inducing glucose intolerance and reducing insulin secretion. We demonstrate that aripiprazole modulates the serotonergic system in islets, increasing mTOR/S6 phosphorylation, as well as elevating TPH1 expression and serotonin production in beta cells. By contrast, the effects of olanzapine on insulin secretion seem to be independent of the serotonergic system. Since type 2 diabetes develops gradually through life, and chronic medication is needed to tackle schizophrenia, we analysed the metabolic disturbances in female mice treated with these two chemically unrelated SGAs via supplementation in the diet over 6 months. To our knowledge, this is the first preclinical study in rodents to report the metabolic outcomes of long-term administration of olanzapine and aripiprazole that focuses on islet function.

Olanzapine treatment induced weight gain by increasing food intake; this finding is in agreement with previous studies that also demonstrated that this effect was mediated by the 5-HT_2C_ [26] and H_1_R receptors in the hypothalamus [27]. Conversely, aripiprazole treatment results in less weight gain and this was not associated with increased food intake, but rather with reduced physical activity and EE during the dark phase, an effect likely contributing to weight gain. Moreover, the effects of olanzapine on EE and physical activity were small, contrary to previous findings [28]. Of clinical relevance, olanzapine-induced weight gain has been reported in patients treated for longer than 12 months, but this has not been the case for aripiprazole [29]. Yet, recent findings point to a mean 6–7% gain in body weight in young people receiving aripiprazole [30]. Remarkably, visceral adiposity was increased in mice treated with either drug, although the effect with aripiprazole treatment was more robust. In studies of olanzapine therapy, increased adiposity has been reported both concomitantly with [31], and also independently from [32] weight gain. Altogether, our results suggest that both olanzapine and aripiprazole increase adiposity, irrespective of the degree of weight gain. Of note, as schizophrenia, per se, concurs with metabolic derangements [33], the metabolic side effects of SGAs are likely to be more severe in the context of this disease. Also, although female patients are more susceptible to changes in glucose metabolism following SGA exposure [6], the current study is a single-sex study, a limitation that needs to be considered for its translatability.

Female mice treated with olanzapine or aripiprazole developed glucose intolerance that was associated with insulin resistance in aripiprazole-treated mice. However, we cannot exclude that longer treatments or more sensitive assays to assess insulin sensitivity, such as the euglycaemic–hyperinsulinaemic clamp, would reveal greater effects on blood glucose levels and insulin sensitivity. A step further, this is the first study to unravel a unique effect of aripiprazole in interfering with glucose-regulated Ca^2+^ signalling, whereas olanzapine likely inhibits insulin secretion through a mechanism distal to Ca^2+^ entry into the beta cell. Of interest, while the GSIS test addresses insulin secretion exclusively, we cannot exclude alterations in hepatic insulin clearance or the beta cell insulin-degrading enzyme, both of which impair insulin secretion [34].

Notably, mice treated with the SGAs had larger islets, particularly the aripiprazole-treated group, in which beta cell mass was twofold higher than that of the control group. In obesity and pregnancy, beta cell expansion is associated with enhanced insulin secretion, which compensates for insulin resistance. On the contrary, aripiprazole impairs insulin secretion despite beta cell expansion, indicating that increased beta cell mass, per se, is not sufficient to overcome beta cell dysfunction. The apparent paradoxical effects on mass and function were more prominent in aripiprazole-treated mice in which doubling of beta cell mass was associated with blunted insulin response.

Treatment with SGAs did not affect beta cell proliferation, which remained low, as previously reported in middle-aged mice [20]. However, a compensatory proliferative response might be expected at an earlier stage of the treatment. On the contrary, we found increased beta cell size in islets from both groups of SGA-treated mice, as compared with controls, which might explain the islet size expansion observed at the end of the treatment. Activation of mTORC1 signalling, which increases islet hypertrophy, has been suggested to be involved in the compensatory beta cell expansion during insulin resistance [35]. Our results showed islet hypertrophy in aripiprazole-treated mice together with increased p-S6 staining in beta cells, an effect reinforced by increased phosphorylation of mTOR and its downstream targets S6K1 and S6 in islets treated ex vivo with this SGA. Thus, the increased islet size and beta cell mass by aripiprazole might be mediated via mTORC1/S6. By contrast, S6 phosphorylation was not increased by olanzapine. At the molecular level, the differential ability of each drug to induce mTORC1/S6 activity or, alternatively, other mechanisms, such as the Hippo pathway [36], might also be implicated in the islet hypertrophy observed with olanzapine. Also, lower mTORC1 activation in olanzapine-treated mice could be due to a more subtle (non-significant) increase in insulin intolerance. It is noteworthy that we found greater (although not significant) differences in insulin granule maturation in olanzapine-treated mice vs the control group, manifested by a decrease and increase in mature and empty granules, respectively (both *p*=0.09), which deserves further investigation. Additionally, olanzapine-treated mice had higher alpha cell mass, pointing to potential pancreatic alterations beyond beta cells.

The complexity of the dopaminergic and serotonergic systems in pancreatic islets, which regulate insulin secretion [10, 37], together with the broad spectrum of dopamine/serotonin receptors targeted by SGAs, makes it difficult to determine whether a specific receptor mediates the effects of a particular SGA or if the final outcome results from signalling pathways activated by multiple receptors. Transcriptomic analysis of pancreatic islets did not show changes in genes related to dopamine signalling, but revealed changes in genes regulating serotonin synthesis. Aripiprazole upregulated *Tph1* and *Tph2* genes, and the induction of *Tph1* mRNA and TPH1 protein levels (the rate-limiting isoform for serotonin biosynthesis) was associated with increased serotonin content and release in islets from aripiprazole-treated mice. These results were supported by: (1) the ex vivo treatment of islets with aripiprazole, which similarly resulted in increased TPH1 expression; (2) the decrease in insulin secretion in islets treated with serotonin that, like aripiprazole, activated mTORC1/S6 signalling; (3) and the recovery of GSIS in islets treated ex vivo with aripiprazole together with a TPH1 inhibitor, pointing to serotonin-mediated inhibition of insulin secretion by this SGA. Our findings are in agreement with a recent study showing that Sirtuin 3 deficiency in beta cells increased *Tph1* expression, along with impairment of GSIS in obese mice [38].

Transcriptomic analysis also showed that olanzapine downregulated the expression of *Htr3a*, which encodes a serotonin receptor, in islets, potentially playing a role in the impairment of insulin secretion by this SGA, as previously reported [39, 40]. Notably, changes in serotonin receptor expression were found in *db/db* mice, which exhibited increased expression of *Htr2c* [41]. So far, the role of serotonin signalling in beta cell expansion has been described only in pregnancy [22, 23] and the perinatal period [42]. Recent studies suggest that increased serotonin production could affect whole-body glucose homeostasis and adiposity [43]. In the context of tumour growth, serotonin increases mTORC1 activity in hepatocellular carcinoma [44], reinforcing a possible link between serotonin and mTORC1/S6 signalling. Because serotonin is also a strong paracrine regulator of alpha cell activity [45], additional effects of aripiprazole on alpha cells functionality cannot be ruled out.

In summary, we have identified alterations in islet plasticity and insulin secretion in female mice treated with the SGAs olanzapine and aripiprazole, with important translational implications. In the case of aripiprazole, in which the serotonergic system was activated, specific TPH1 inhibitors that do not cross the blood–brain barrier could be used to prevent intra-islet and peripheral serotonin dysregulation without affecting serotonin levels in the brain [46].

## Supplementary information

ESM (PDF 902 kb)

## Data Availability

Data presented in this manuscript are available upon request from the corresponding author.

## Abbreviations

D2R: Dopamine D2 receptor
D3R: Dopamine D3 receptor
D4R: Dopamine D4 receptor
DEGs: Differentially expressed genes
EE: Energy expenditure
ΔF: Change in fluorescence
F_basal_: Basal fluorescence
GSIS: Glucose-stimulated insulin secretion
H_1_R: Histamine H_1_ receptor
5-HT: 5-Hydroxytryptamine
iWAT: Inguinal white adipose tissue
M1R: Muscarinic M1 receptor
M5R: Muscarinic M5 receptor
mTOR: Mechanistic target of rapamycin
mTORC1: Mechanistic target of rapamycin complex 1
ORA: Over-representation analysis
*p*-adj: Adjusted *p* value
PCA: Principal component analyses
PCPA: 4-Chloro-dl-phenylalanine
RER: Respiratory exchange ratio
RNA-seq: RNA-sequencing
RT-qPCR: Quantitative real-tim**e** PCR
SGA: Second-generation antipsychotics
TEM: Transmission electron microscopy
TPH1: Tryptophan hydroxylase 1
WAT: White adipose tissue
