# Supplementary material for: The second-generation antipsychotic drug aripiprazole modulates the serotonergic system in pancreatic islets and induces beta cell dysfunction in female mice
Source: Diabetologia. 2021 Dec 21;65(3):490–505. doi: 10.1007/s00125-021-05630-0 (PMC8803721; doi:10.1007/s00125-021-05630-0)
Supplement: Supplementary file 1 — (PDF 902 kb) [file 125_2021_5630_MOESM1_ESM.pdf]

Electronic Supplementary Material (ESM)

ESM Methods

Analysis of olanzapine and aripiprazole in plasma

A simple and sensitive liquid chromatography-tandem mass spectrometry (LC-MS/MS) method was used for the simultaneous determination of aripiprazole and olanzapine. The above-mentioned compounds and their isotope-labeled internal standards were extracted from 100 µl of plasma by protein precipitation. A combination of formic acid (0.2%)-acetonitrile (pH 3.0; 65:35, vol./vol.) was used as mobile phase and the chromatogram was run under gradient conditions at a flow rate of 0.6 ml/min. Run time lasted 6 min, followed by a re-equilibration time of 3 min. All analytes were monitored by mass spectrometric detection operating in multiple-reaction monitoring mode. The method was originally validated in human plasma based on the recommendations of regulatory agencies through tests of precision, accuracy, extraction recovery, identity confirmation, trueness, matrix effect, process efficiency, stability, selectivity, linearity and carry-over effect fulfilling the guideline requirements.

In vivo GTT, ITT and GSIS

After 6 months on diet, metabolic assays were performed in the experimental groups. In the GTT and GSIS tests, glucose (2 g/kg) was injected intraperitoneally (i.p.) in mice after 16 h of fasting at 8:00 AM. Blood samples were collected from the tail vein at 0, 5, 10, 15, 30, 60 and 120 min. Plasma glucose and insulin levels were measured with a glucometer and a mouse insulin ELISA (10-1247-01, Mercodia, Sweden), respectively. In the ITT, mice were fasted for 4 h and human recombinant insulin (0.75 U/kg; Actrapid; Novo Nordisk, Denmark) was i.p. injected at 12:00 PM. Blood glucose was measured at 0, 15, 30, 60 and 90 min post-injection.

Indirect calorimetry

Indirect calorimetry analysis was carried out at the end of the study using the TSE Phenomaster monitoring system (TSE Systems GmbH, Germany). Mice were placed at the room where the equipment was located 4 days before the analysis and distributed in individual measuring cages for at least 24 h prior to data recording. Mice had full access to food and water and calorimetry measurements were carried out during a period of 72 h. Mice were on a 12-hour light-dark cycle and room temperature was maintained at 22 ± 2ºC. Oxygen consumption and the release of CO_2 were measured. From these values, respiratory exchange ratio (RER) was determined as VCO_2/O_2 and energy expenditure was calculated as $EE = (3.185 + 1.232 \times RER) \times VO_2$. Total locomotor activity

was simultaneously measured on the X and the Y axes using an infrared photocell beam interruption method as described [1]. Analysis was performed using the TSE Phenomaster Mouse software V5.1.7 (TSE Systems GmbH, Germany).

Morphometric analysis of the pancreas

To determine alpha and beta cell mass, longitudinal pancreatic sections of 6 µm thickness generated every 80 µm spanning the entire pancreas were stained for glucagon and insulin, respectively. Images were examined using a Axiophot Zeiss light microscope (Zeiss, Germany) and captured with a DP70 digital camera (Olympus, Japan) at an original magnification of x 4. The percent area covered by insulin or glucagon and the total pancreatic area were quantified by the Image J software (NIH). Alpha and beta cell mass (in mg) were calculated as the product of pancreatic weight (in mg) and the fractional alpha or beta cell area (%) in different sections, respectively.

Immunofluorescence of pancreatic sections

Pancreatic sections rehydrated and processed for antigen retrieval were incubated with primary antibodies against insulin, glucagon, Ki67, serotonin and phospho-S6 ribosomal protein diluted in PBS-3% BSA (wt/vol.) o/n at 4ºC. Antibodies are detailed in ESM Table 1 and were validated following manufacturer´s instructions. After washing, sections were incubated with secondary antibodies coupled to fluorophores in PBS with DAPI (#D1306; 1:10000, ThermoFisher Scientific, USA) for 1 h at RT. Secondary antibodies used are listed in ESM Table 1. Immunofluorescence was examined using an epifluorescence microscope (Nikon 90i, Olympus) and images were taken with a digital camera (Nikon DS-2Mv, Japan). Beta cell proliferation, serotonin and phospho-S6 expression were measured by the percentage of insulin and Ki67, insulin and serotonin or insulin and phospho-S6 co-positive cells in all beta cells.

Immunofluorescence of pancreatic islets

Twenty islets, treated for 24 h as described in the previous sections, were handpicked with a 10 µl pipette and placed in µ-slide 8-well plates (80826; Ibidi, Germany). Islets were washed with PBS and permeabilized with 1% (vol./vol.) Triton-X in PBS for 30 min. Thereafter, islets were incubated with blocking solution containing 3% (vol./vol.) normal donkey serum in 3% BSA (wt/vol) in PBS for 1 h and then incubated at 4ºC overnight with the primary antibodies diluted in blocking solution. Primary antibodies against insulin and glucagon were used (ESM Table 1). The day after, islets were washed and incubated with secondary antibodies coupled to fluorophores in PBS with DAPI

(#D1306; 1:10000, ThermoFisher Scientific, USA) for 1 h at RT. Secondary antibodies used are in ESM Table 1. All antibodies were validated following manufacturer's instructions. After washing with PBS, islets were mounted in the µ-slide 8-well plate with Prolong diamond antifade mounting medium (P36961; ThermoFisher Scientific, USA). Immunofluorescence was examined using an epifluorescence microscope (Nikon 90i, Olympus) and images were taken with a digital camera (Nikon DS-2Mv, Japan).

Ultrastructural analysis by Transmission Electron Microscopy (TEM)

For Transmission Electron Microscopy (TEM) analysis, pools of 300 pancreatic islets from 3 mice per group were processed using a standard Spurr protocol. Islets were isolated and washed in cold PBS and thereafter fixed for 4 h at 4ºC in 2.5% (vol./vol.) glutaraldehyde solution prepared in 0.1 mol/l sodium cacodylated buffer, pH 7.4. After washing, samples were post fixed for 1 h at 4ºC in 2% OsO_4 (wt/vol.) prepared in 0.1 mol/l sodium cacodylated buffer, pH 7.4. Thereafter the samples were immersed in 2% (wt/vol.) uranyl acetate, dehydrated through a gradient acetone series (50%, 70%, 90% and 100%, vol./vol.) and embedded in Spurr resin (Electron Microscopy Science, USA). Blocks were obtained by polymerization at 70ºC for 8 h. Thin sections were cut with a diamond knife in an ultramicrotome (Leica UC7; Leica Biosystems, Germany) and examined with a transmission electron microscope (Zeiss Libra 120) operating at 80 kV. TEM images were taken at different magnifications with an EMCCD camera (TRS 2k x 2k) (Albert Tröndle, Germany). We quantified the secretory granules of 10 beta cells from 3 independent mice per group. We classified them into four different categories: mature, those having an electron-dense core; immature, those having a less electron-dense core; empty, lacking the core, or atypical, those insulin granules with irregular shape.

Protein extraction and Western blotting

Pancreatic islets were lysed in Cell Lysis Buffer (#9803, Cell Signaling, USA) or lysis buffer containing 10 mmol/l Tris pH 7.5, 5 mmol/l EDTA, 50 mmol/l HCl, 30 µmol/l sodium pyrophosphate, 50 mmol/l NaF, 100 mmol/l o-vanadate sodium, 1% (vol./vol.) Triton X-100, 1 mmol/l phenylmethylsulfonyl fluoride (PMSF) and 10 µg/ml of protease inhibitors (P8340; Sigma-Aldrich, USA), pH 7.4-7.6. Protein extracts (15-30 µg) were loaded in 8-12% SDS polyacrylamide gel electrophoresis (SDS-PAGE) and blotted (PVDF membranes, IPVH00010; Merck). Membranes were incubated in blocking solution (5% (wt/vol.) skimmed milk in Tris-buffered saline pH 7.5 containing 0.05% (vol./vol.) Tween-20) for 1 h at RT followed by overnight incubation (4ºC) with primary antibodies. The day after, blots were incubated with secondary antibodies. Primary and secondary antibodies are listed in ESM Table 1 and were validated following manufacturer´s instructions.

Immunoreactivity was detected by chemiluminescence. Densitometric analysis of the bands was performed using the Image J software (NIH, USA).

Quantitative real-time PCR (RT-qPCR)

Gene expression was determined by quantitative RT-qPCR on a 7900HT Fast Real-Time PCR System (ThermoFisher Scientific, USA) with SYBR Green (Thermofisher Scientific, USA) detection protocol. Samples were run in duplicate for each assayed gene, and presented as the fold change in gene expression normalized to the endogenous reference genes (*Gapdh* and *Actb*) and relative to the control condition ($-2^{\Delta\Delta Ct}$ method). Primer sequences are listed in ESM Table 2.

ESM Tables

ESM Table 1. Antibodies.

	Manufacturer	Catalog number	Host species	Dilution
Glucagon	LINCO Research INC	4031-01F	Guinea pig	1:500 (immunohistochemistry)
	DAKO, Argilent	A0565	Rabbit	1:300 (immunofluorescence)
Insulin	Abcam	Ab7842	Guinea pig	1:100 (immunohistochemistry)<br>1:50 (immunofluorescence)
IRS-2	Cell Signaling	4502	Rabbit	1:100 (western blot)
Ki67	Abcam	Ab16667	Rabbit	1:100 (immunofluorescence)
mTOR	Cell Signaling	2983	Rabbit	1:1000 (western blot)
p-mTOR (Ser2448)	Cell Signaling	2971	Rabbit	1:1000 (western blot)
p-S6 (Ser240/244)	Cell Signaling	2215	Rabbit	1:100 (immunofluorescence)
S6K1	Cell Signaling	2708	Rabbit	1:1000 (western blot)
p-S6K1 (Thr389)	Cell Signaling	9234	Rabbit	1:1000 (western blot)
Serotonin	Immunostar	20080	Rabbit	1:100 (immunofluorescence)
TPH1	Invitrogen	PA1-777	Rabbit	1:1000 (western blot)
Vinculin	Santa Cruz Biotechnology	Sc-73614	Mouse	1:15000 (western blot)

Biotinylated anti-guinea pig	Vector Laboratories	BA-7000	Goat	1:250 (immunohistochemistry)
Alexa Fluor-conjugated secondary antibodies	ThermoFisher Scientific	A-21435	Goat	1:250 (immunofluorescence)
		A-11034	Goat	1:250 (immunofluorescence)
Secondary anti-mouse	Santa Cruz Biotechnology	Sc-2005	Goat	1:10000 (western blot)
Secondary anti-rabbit	Bethyl Laboratories Inc	A120-108P	Goat	1:25000 (western blot)

ESM Table 2. Primers used in RT-qPCR.

**Gene**	**Specie**	**Forward primer (5'→3')**	**Reverse primer (5'→3')**
Actb	Mouse	AGGAGGAGCAATGATCTTGATCTT	TCCTTCCTGGGCATGGAG
Gcg	Mouse	CCAAATCAAGGGATAAGACCCTC	AAGCTCTGCCCTTCTGCACCAG
Gck	Mouse	GTGATAGGCACCAAGGCACTGAC	CGGTGCTTCTGTTCCAACCAGG
Gapdh	Mouse	AGGTCGGTGTGAACGGATTTG	TGTAGACCATGTAGTTGAGGTCA
Htr2b	Mouse	GAACAAAGCACAACTTCTGAGC	CCGCGAGTATCAGGAGAGC
Htr2c	Mouse	GTTCAATTCGCGGACTAAGG	TCACGAACACTTTGCTTTCG
Htr3a	Mouse	CCTGGCTAACTACAAGAAGGGG	TGCAGAAACTCATCAGTCCAGTA
Ins-1	Mouse	TAGTGACCAGCTATAATCAGA	AACGCCAAGGTCTGAAGGTCC
Ins-2	Mouse	CCCTGCTGGCCCTGCTCTT	GGTCTGAAGGTCACCTGCT
Pdx1	Mouse	GAACCCGAGGAAAACAAGAGG	GTTCAACATCACTGCCAGCTC
Tph1	Mouse	GACCATCTTCCGAGAGCTAAACAA	AGCAAAGGGAGGTTTCTGAGG TA
Tph2	Mouse	TGGCAAAACGGAATTCAA	CCTCCGTCCAAATGCTCTCA

ESM Table 3. Differentially expressed genes in islets from olanzapine vs chow diet-fed mice.

Ensembl ID	Gene symbol	padj	log2FoldChange
ENSMUSG00000048126	*Col6a3*	9.1E-05	2,01
ENSMUSG00000004655	*Aqp1*	5.1E-04	-3.91
ENSMUSG00000021676	*Iqgap2*	5.1E-04	1.73
ENSMUSG00000054545	*Ugt1a6a*	1.1E-03	1.52
ENSMUSG00000040170	*Fmo2*	3.3E-02	0.96
ENSMUSG00000054000	*Tusc1*	3.3E-02	1.73
ENSMUSG00000026347	*Tmem163*	3.6E-02	-0.73
ENSMUSG00000024293	*Esco1*	3.6E-02	0.52
ENSMUSG00000027829	*Ccnl1*	3.6E-02	0.61
ENSMUSG00000070713	*Gm10282*	3.6E-02	2.02
ENSMUSG00000114133	*Gm20075*	7.1E-02	3.64
ENSMUSG00000022297	*Fzd6*	8.2E-02	1.49
ENSMUSG00000107137	*Fzd6*	8.2E-02	1.49
ENSMUSG00000021069	*Pygl*	9.6E-02	-0.83
ENSMUSG00000022797	*Tfrc*	9.9E-02	0.50

ESM Table 4. Differentially expressed genes in islets from aripiprazole vs chow diet-fed mice.

Ensembl ID	Gene symbol	padj	log2FoldChange
ENSMUSG00000025716	*Myo3a*	5.66E-07	2.17
ENSMUSG00000022324	*Matn2*	5.66E-07	2.27
ENSMUSG00000030075	*Cntn3*	2.39E-06	1.27
ENSMUSG00000022425	*Enpp2*	2.39E-06	1.34
ENSMUSG00000021730	*Hcn1*	1.10E-05	2.87
ENSMUSG00000024411	*Aqp4*	1.40E-05	1.51
ENSMUSG00000040181	*Fmo1*	3.22E-05	2.78
ENSMUSG00000004655	*Aqp1*	4.54E-05	-4.51
ENSMUSG00000043300	*B3galnt1*	4.54E-05	1.82
ENSMUSG00000019990	*Pde7b*	1.36E-04	1.32
ENSMUSG00000021596	*Mctp1*	1.66E-04	2.09
ENSMUSG00000006764	*Tph2*	1.91E-04	2.56
ENSMUSG00000047631	*Apof*	3.68E-04	-2.70
ENSMUSG00000040170	*Fmo2*	6.20E-04	1.11

ENSMUSG00000008206	*Cers4*	6.99E-04	-0.94
ENSMUSG00000020671	*Rab10*	8.54E-04	0.74
ENSMUSG00000029265	*Dr1*	8.54E-04	0.99
ENSMUSG00000035910	*Dcdc2a*	1.15E-03	-0.90
ENSMUSG00000034701	*Neurod1*	1.79E-03	1.14
ENSMUSG00000070713	*Gm10282*	1.95E-03	2.21
ENSMUSG00000027350	*Chgb*	2.04E-03	0.94
ENSMUSG00000041261	*Car8*	2.05E-03	1.35
ENSMUSG00000078887	*Gm6710*	2.45E-03	1.04
ENSMUSG00000026312	*Cdh7*	2.55E-03	0.80
ENSMUSG00000092021	*Gbp11*	4.05E-03	4.13
ENSMUSG00000021720	*Rnf180*	4.10E-03	1.13
ENSMUSG00000032263	*Bckdhb*	4.33E-03	0.73
ENSMUSG00000114133	*Gm20075*	4.73E-03	3.96
ENSMUSG00000025937	*Lactb2*	4.96E-03	0.78
ENSMUSG00000027875	*Hmgcs2*	5.64E-03	-4.70
ENSMUSG00000016239	*Lonrf3*	5.64E-03	1.66
ENSMUSG00000030134	*Rasgef1a*	6.22E-03	-1.44
ENSMUSG00000040724	*Kcna2*	6.24E-03	1.13
ENSMUSG00000021379	*Id4*	6.82E-03	2.39
ENSMUSG00000117234	*Gm7818*	8.60E-03	2.18
ENSMUSG00000050069	*Grem2*	1.07E-02	1.82
ENSMUSG00000079084	*Ccdc82*	1.24E-02	0.71
ENSMUSG00000037379	*Spon2*	1.28E-02	-0.76
ENSMUSG00000036880	*Acaa2*	1.38E-02	-0.83
ENSMUSG00000026072	*Il1r1*	1.47E-02	-0.61
ENSMUSG00000075316	*Scn9a*	1.47E-02	0.62
ENSMUSG00000014905	*Dnajb9*	1.47E-02	1.05
ENSMUSG00000062275	*Fbxw24*	1.50E-02	2.90
ENSMUSG00000081683	*Fzd10*	1.52E-02	-2.91
ENSMUSG00000054545	*Ugt1a6a*	1.69E-02	1.22
ENSMUSG00000036887	*C1qa*	1.73E-02	0.90
ENSMUSG00000068263	*Efcc1*	1.79E-02	-1.32
ENSMUSG00000035851	*Ythdc1*	1.80E-02	0.55
ENSMUSG00000004791	*Pgf*	1.86E-02	-0.95
ENSMUSG00000024187	*Fam234a*	1.87E-02	-0.68

ENSMUSG00000028042	*Zbtb7b*	1.87E-02	-0.67
ENSMUSG00000109925	*Gm45315*	1.89E-02	-2.05
ENSMUSG00000042901	*Aida*	1.89E-02	0.66
ENSMUSG00000051412	*Vamp7*	1.89E-02	1.22
ENSMUSG00000022297	*Fzd6*	1.89E-02	1.51
ENSMUSG00000107137	*Fzd6*	1.89E-02	1.51
ENSMUSG00000027523	*Gnas*	1.93E-02	-0.48
ENSMUSG00000002504	*Slc9a3r2*	1.94E-02	-0.88
ENSMUSG00000050520	*Cldn8*	1.94E-02	2.87
ENSMUSG00000020246	*Hcfc2*	2.19E-02	0.82
ENSMUSG00000080242	*Atp6v0c-ps2*	2.21E-02	-1.94
ENSMUSG00000060935	*Tmem263*	2.21E-02	0.79
ENSMUSG00000039899	*Fgl2*	2.21E-02	0.85
ENSMUSG00000073079	*Srp54a*	2.21E-02	0.88
ENSMUSG00000027400	*Pdyn*	2.21E-02	1.13
ENSMUSG00000017204	*Gsdma*	2.21E-02	1.57
ENSMUSG00000026938	*Fcna*	2.22E-02	-3.10
ENSMUSG00000058076	*Sdhc*	2.22E-02	-0.88
ENSMUSG00000038267	*Slc22a23*	2.22E-02	-0.69
ENSMUSG00000037579	*Kcnh3*	2.48E-02	-1.66
ENSMUSG00000058600	*Rpl30*	2.52E-02	0.91
ENSMUSG00000079108	*Srp54c*	2.72E-02	0.85
ENSMUSG00000024078	*Ttc27*	3.15E-02	-0.60
ENSMUSG00000096433	*Zfp994*	3.15E-02	0.96
ENSMUSG00000036699	*Zcchc12*	3.15E-02	0.97
ENSMUSG00000036095	*Dgkb*	3.17E-02	0.87
ENSMUSG00000019929	*Dcn*	3.17E-02	-1.80
ENSMUSG00000029368	*Alb*	3.37E-02	-6.15
ENSMUSG00000059136	*Olfr539*	3.37E-02	-1.14
ENSMUSG00000047213	*Ythdf3*	3.37E-02	0.51
ENSMUSG00000040297	*Suco*	3.37E-02	0.65
ENSMUSG00000031825	*Crispld2*	3.37E-02	4.05
ENSMUSG00000028005	*Gucy1b1*	3.40E-02	-0.79
ENSMUSG00000026787	*Gad2*	3.40E-02	1.97
ENSMUSG00000026692	*Fmo4*	3.41E-02	1.38
ENSMUSG00000044501	*Zfp758*	3.67E-02	1.03

ENSMUSG00000030532	*Hddc3*	3.73E-02	0.69
ENSMUSG00000030659	*Nucb2*	3.73E-02	0.70
ENSMUSG00000049241	*Hcar1*	3.88E-02	-1.36
ENSMUSG00000070003	*Ssbp4*	3.88E-02	-1.03
ENSMUSG00000024665	*Fads2*	3.88E-02	-0.86
ENSMUSG00000034205	*Loxl2*	3.88E-02	-0.81
ENSMUSG00000037706	*Cd81*	3.88E-02	-0.56
ENSMUSG00000096696	*Zfp960*	3.88E-02	0.74
ENSMUSG00000031367	*Ap1s2*	3.88E-02	0.78
ENSMUSG00000026950	*Neb*	3.88E-02	1.29
ENSMUSG00000054414	*Slc30a7*	4.08E-02	0.49
ENSMUSG00000048677	*Tpcn2*	4.12E-02	-0.99
ENSMUSG00000030222	*Rerg*	4.12E-02	3.16
ENSMUSG00000024186	*Rgs11*	4.19E-02	-0.70
ENSMUSG00000043587	*Pxylp1*	4.32E-02	-0.82
ENSMUSG00000094526	*Gm21451*	4.47E-02	-3.01
ENSMUSG00000001901	*Kcnh6*	4.47E-02	-0.79
ENSMUSG00000024168	*Tmem204*	4.47E-02	-0.67
ENSMUSG00000053291	*Rab4b*	4.56E-02	-0.64
ENSMUSG00000038793	*Lefty1*	4.70E-02	-0.99
ENSMUSG00000063558	*Aox1*	4.77E-02	0.56
ENSMUSG00000037106	*Fer1l6*	4.77E-02	0.71
ENSMUSG00000116271	*Fer1l6*	4.77E-02	0.71
ENSMUSG00000031107	*Rbmx2*	4.81E-02	1.03
ENSMUSG00000031683	*Lsm6*	4.85E-02	0.86
ENSMUSG00000094388	*Gm8783*	4.96E-02	-2.33
ENSMUSG00000096592	*Gm15801*	4.96E-02	-2.33
ENSMUSG00000052581	*Lrrtm4*	4.96E-02	1.06
ENSMUSG00000036585	*Fgf1*	5.13E-02	-1.06
ENSMUSG00000037458	*Azin1*	5.28E-02	0.59
ENSMUSG00000086133	*Gm16331*	5.28E-02	1.51
ENSMUSG00000040046	*Tph1*	5.37E-02	3.46
ENSMUSG00000023186	*Vwa5a*	5.43E-02	0.52
ENSMUSG00000049265	*Kcnk3*	5.53E-02	-1.20
ENSMUSG00000036502	*Tmem255a*	5.53E-02	0.97
ENSMUSG00000037279	*Ovol2*	5.53E-02	1.10

ENSMUSG00000031202	*Rab39b*	5.59E-02	0.89
ENSMUSG00000029131	*Dnajb6*	5.78E-02	0.51
ENSMUSG00000032328	*Tmem30a*	5.93E-02	0.80
ENSMUSG00000020866	*Cacna1g*	6.01E-02	-2.19
ENSMUSG00000027479	*Mapre1*	6.01E-02	0.85
ENSMUSG00000027582	*Zgpat*	6.30E-02	-0.58
ENSMUSG00000026814	*Eng*	6.40E-02	-0.97
ENSMUSG00000042453	*Reln*	6.60E-02	-0.97
ENSMUSG00000037475	*Thoc2*	6.60E-02	0.56
ENSMUSG00000068270	*Shroom4*	6.64E-02	-0.75
ENSMUSG00000058248	*Kcnh1*	6.72E-02	-0.75
ENSMUSG00000027828	*Ssr3*	6.72E-02	0.72
ENSMUSG00000000317	*Bcl6b*	6.76E-02	0.68
ENSMUSG00000057455	*Rit2*	6.84E-02	0.94
ENSMUSG00000022033	*Pbk*	6.93E-02	2.06
ENSMUSG00000033470	*Cysltr2*	6.93E-02	-1.69
ENSMUSG00000060591	*Ifitm2*	6.93E-02	-0.80
ENSMUSG00000049313	*Sorl1*	6.93E-02	0.53
ENSMUSG00000021703	*Serinc5*	6.93E-02	0.62
ENSMUSG00000055639	*Dach1*	6.94E-02	0.57
ENSMUSG00000026728	*Vim*	6.94E-02	-1.17
ENSMUSG00000023140	*Reg2*	7.30E-02	-2.77
ENSMUSG00000044912	*Syt16*	7.32E-02	-0.94
ENSMUSG00000040836	*Gpr161*	7.32E-02	-0.61
ENSMUSG00000017057	*Il13ra1*	7.32E-02	0.76
ENSMUSG00000064360	*mt-Nd3*	7.32E-02	1.77
ENSMUSG00000069972	*Rps13-ps2*	7.40E-02	9.36
ENSMUSG00000038515	*Grtp1*	7.72E-02	-0.62
ENSMUSG00000061665	*Cd2ap*	7.72E-02	0.46
ENSMUSG00000059921	*Unc5c*	7.88E-02	-0.66
ENSMUSG00000062078	*Qk*	8.01E-02	-0.58
ENSMUSG00000030256	*Bhlhe41*	8.01E-02	0.53
ENSMUSG00000079659	*Tmem243*	8.01E-02	0.58
ENSMUSG00000030704	*Rab6a*	8.01E-02	0.58
ENSMUSG00000041773	*Enc1*	8.01E-02	0.75
ENSMUSG00000059839	*Zfp874b*	8.01E-02	0.76

ENSMUSG00000026834	*Acvr1c*	8.01E-02	0.82
ENSMUSG00000023915	*Tnfrsf21*	8.01E-02	0.84
ENSMUSG00000063412	*Gm10131*	8.01E-02	1.78
ENSMUSG00000015468	*Notch4*	8.19E-02	-1.10
ENSMUSG00000029270	*Dipk1a*	8.19E-02	1.13
ENSMUSG00000027274	*Mkks*	8.25E-02	0.71
ENSMUSG00000034438	*Gbp8*	8.25E-02	1.49
ENSMUSG00000083833	*Gm13841*	8.29E-02	-2.71
ENSMUSG00000094497	*Gm8210*	8.29E-02	-2.71
ENSMUSG00000026768	*Itga8*	8.29E-02	-1.92
ENSMUSG00000026921	*Egfl7*	8.29E-02	-0.68
ENSMUSG00000024583	*Txnl1*	8.29E-02	0.64
ENSMUSG00000056596	*Trnp1*	8.29E-02	1.90
ENSMUSG00000043719	*Col6a6*	8.34E-02	1.00
ENSMUSG00000040714	*Klc3*	8.42E-02	-0.93
ENSMUSG00000068876	*Cgn*	8.42E-02	-0.48
ENSMUSG00000030660	*Pik3c2a*	8.42E-02	0.71
ENSMUSG00000079037	*Prnp*	8.42E-02	0.76
ENSMUSG00000040613	*Apobec1*	8.42E-02	0.92
ENSMUSG00000005268	*Prlr*	8.42E-02	1.01
ENSMUSG00000027513	*Pck1*	8.50E-02	-3.46
ENSMUSG00000023266	*Frs3*	8.50E-02	-1.35
ENSMUSG00000028292	*Rars2*	8.50E-02	0.49
ENSMUSG00000063273	*Naa15*	8.50E-02	0.63
ENSMUSG00000090698	*Apold1*	8.50E-02	0.64
ENSMUSG00000053347	*Zfp943*	8.50E-02	0.67
ENSMUSG00000068130	*Zfp442*	8.50E-02	0.68
ENSMUSG00000026761	*Orc4*	8.50E-02	0.69
ENSMUSG00000020650	*Bcap29*	8.50E-02	0.81
ENSMUSG00000054404	*Slfn5*	8.50E-02	0.82
ENSMUSG00000033972	*Zfp944*	8.50E-02	0.84
ENSMUSG00000060780	*Lrrtm1*	8.55E-02	-1.89
ENSMUSG00000002763	*Pex6*	8.55E-02	-0.65
ENSMUSG00000027304	*Rtf1*	8.55E-02	-0.63
ENSMUSG00000027808	*Serp1*	8.55E-02	0.77
ENSMUSG00000033953	*Ppp3r1*	8.64E-02	0.63

ENSMUSG00000027297	*Ltk*	8.75E-02	-1.19
ENSMUSG00000030055	*Rab43*	8.75E-02	-1.01
ENSMUSG00000029123	*Stk32b*	8.75E-02	1.28
ENSMUSG00000038241	*Cep250*	8.77E-02	-0.74
ENSMUSG00000046668	*Cxxc5*	8.77E-02	-0.63
ENSMUSG00000026254	*Eif4e2*	8.77E-02	-0.51
ENSMUSG00000090093	*Gm14399*	8.77E-02	0.48
ENSMUSG00000048100	*Taf13*	8.77E-02	0.86
ENSMUSG00000032034	*Kcnj5*	8.77E-02	1.39
ENSMUSG00000024952	*Rps6ka4*	8.80E-02	-0.96
ENSMUSG00000019838	*Slc16a10*	8.83E-02	-0.60
ENSMUSG00000025856	*Pdgfa*	8.92E-02	-0.58
ENSMUSG00000030265	*Kras*	8.92E-02	0.39
ENSMUSG00000022414	*Tab1*	8.95E-02	-0.51
ENSMUSG00000021785	*Ngly1*	9.00E-02	0.51
ENSMUSG00000035206	*Sppl2b*	9.06E-02	-0.90
ENSMUSG00000021587	*Pcsk1*	9.28E-02	0.67
ENSMUSG00000032440	*Tgfbr2*	9.38E-02	-0.68
ENSMUSG00000054408	*Spcs3*	9.38E-02	0.62
ENSMUSG00000044164	*Rnf182*	9.38E-02	1.07
ENSMUSG00000036093	*Arl5a*	9.39E-02	0.60
ENSMUSG00000025326	*Ube3a*	9.44E-02	0.52
ENSMUSG00000025894	*Aasdhppt*	9.44E-02	0.64
ENSMUSG00000040964	*Arhgef10l*	9.49E-02	-1.29
ENSMUSG00000029703	*Lrwd1*	9.49E-02	-0.55
ENSMUSG00000020892	*Aloxe3*	9.49E-02	-1.51
ENSMUSG00000037736	*Limch1*	9.49E-02	-0.63
ENSMUSG00000028132	*Tlcd4*	9.49E-02	0.46
ENSMUSG00000006651	*Aplp1*	9.52E-02	-0.64
ENSMUSG00000031112	*Stk26*	9.54E-02	0.80
ENSMUSG00000009628	*Tex15*	9.54E-02	3.02
ENSMUSG00000025730	*Rab40c*	9.54E-02	-0.89
ENSMUSG00000036815	*Dpp10*	9.54E-02	0.79
ENSMUSG00000057836	*Xlr3a*	9.55E-02	-0.53
ENSMUSG00000063889	*Crem*	9.55E-02	0.73
ENSMUSG00000066551	*Hmgb1*	9.55E-02	0.87

ENSMUSG00000060923	*Acyp2*	9.65E-02	0.89
ENSMUSG00000070368	*Prok1*	9.71E-02	3.25
ENSMUSG00000072115	*Ang*	9.74E-02	-0.70
ENSMUSG00000041120	*Nbl1*	9.76E-02	-0.66
ENSMUSG00000091071	*1700030C10Rik*	9.83E-02	-1.46
ENSMUSG00000038146	*Notch3*	9.83E-02	-0.87
ENSMUSG00000031808	*Slc27a1*	9.83E-02	-0.72
ENSMUSG00000061603	*Akap6*	9.89E-02	-0.88
ENSMUSG00000053166	*Cdh22*	9.89E-02	-0.85
ENSMUSG00000031502	*Col4a1*	9.89E-02	-0.57
ENSMUSG00000027829	*Ccnl1*	9.89E-02	0.46
ENSMUSG00000021427	*Ssr1*	9.89E-02	0.62
ENSMUSG00000029869	*Ephb6*	9.89E-02	1.64
ENSMUSG00000075224	*Lrrc55*	1.00E-01	3.69

ESM Fig. 1

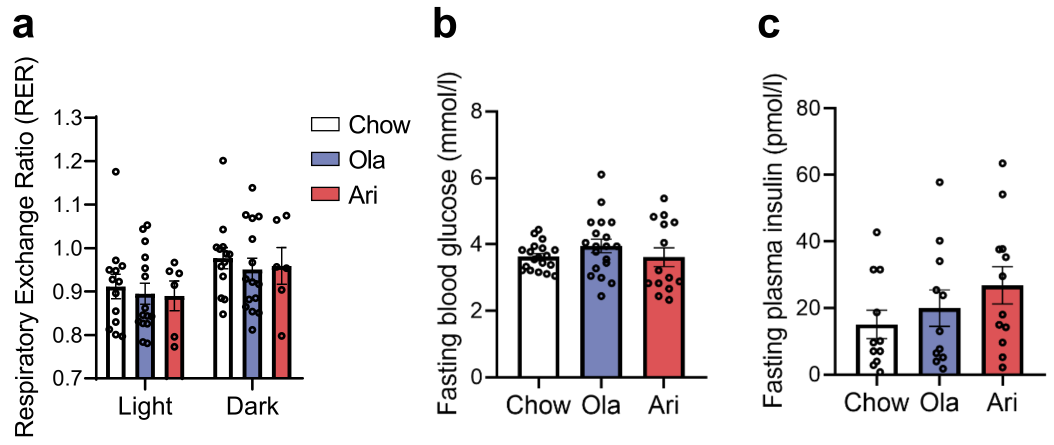

ESM Figure 1. Analysis of RER, glucose and insulin levels in female mice fed an antipsychotic-supplemented diet. **(a)** RER was measured at the end of the treatment by indirect calorimetry (n=6-15). Data are presented as mean ± SEM. Light cycle (8:00 AM – 8:00 PM). Dark cycle (8:00 PM – 8:00 AM). **(b)** Fasting glucose levels (mmol/l) (n=14-19 mice/group. **(c)** Fasting plasma insulin levels (pmol/l) (n=11-12 mice/group). Data are presented as mean ± SEM. P-values were determined by one-way ANOVA and Bonferroni Post-hoc test. No statistical significance was reached between groups.

ESM Fig. 2

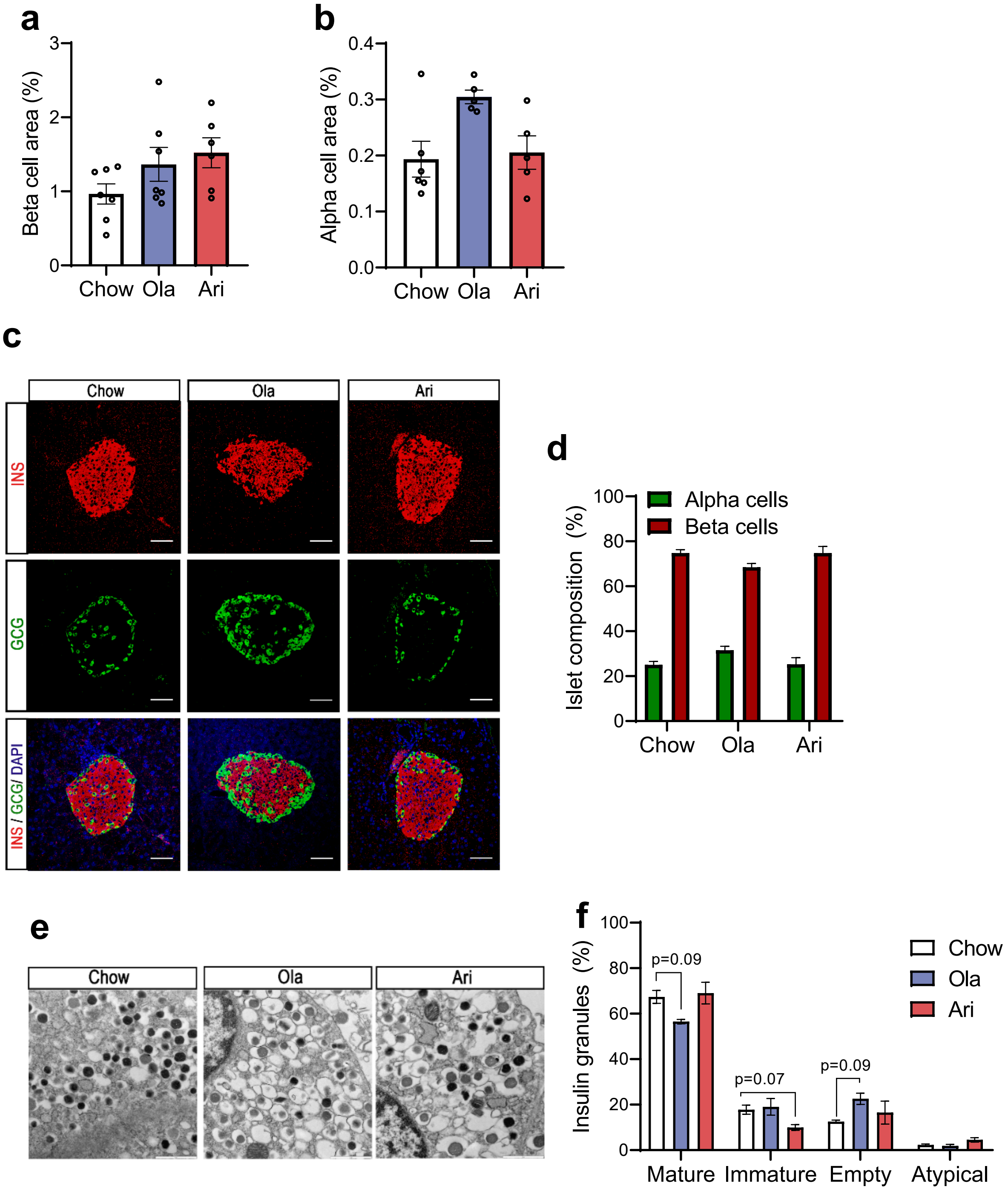

a
Beta cell area (%)
Chow
Ola
Ari
b
Alpha cell area (%)
c
INS
GCG
INS / GCG/ DAPI
d
Alpha cells
Beta cells
Islet composition (%)
e
f
Insulin granules (%)
p=0.09
p=0.07
Mature
Immature
Empty
Atypical

ESM Figure 2. Analysis of alpha and beta cells in islets from female mice fed an antipsychotic-supplemented diet. **(a)** Beta cell area expressed as percentage of the insulin-positive area over the total pancreatic area. **(b)** Alpha cell area expressed as percentage of the glucagon-positive area over the total pancreatic area. Data are presented as mean ± SEM. Between 8 and 12 pancreatic sections per mouse, generated every 80 µm, were analysed. (n=5-6 mice/group). P-values were determined by one-way ANOVA test and Bonferroni Post-hoc test. **(c)** Double immunostaining of pancreatic sections with insulin (red) and glucagon (green). Images were captured by confocal microscopy. Scale bar (40 X) = 50 µm. **(d)** Percentage of alpha and beta cells. Data are presented as mean ± SEM. A total of 25.16 ± 1.24 islets were quantified for alpha and beta cell composition (n= 4 mice/group)**.** Two pancreatic sections per mouse were analysed, with each section being generated every 200 µm. P-values were determined by two-way ANOVA test or Kruskal-Wallis and Bonferroni Post-hoc test. No statistical significance was reached when the three groups of mice were compared. **(e)** Representative TEM images of insulin granules of beta cells from each treatment**.** Scale bar = 1 µm. **(f)** Quantification of insulin granule categories in different groups (n=3 mice/group). Ten cells sections per mouse were analyzed. TEM analysis of the granules did not reach statistical significance.

ESM Fig. 3

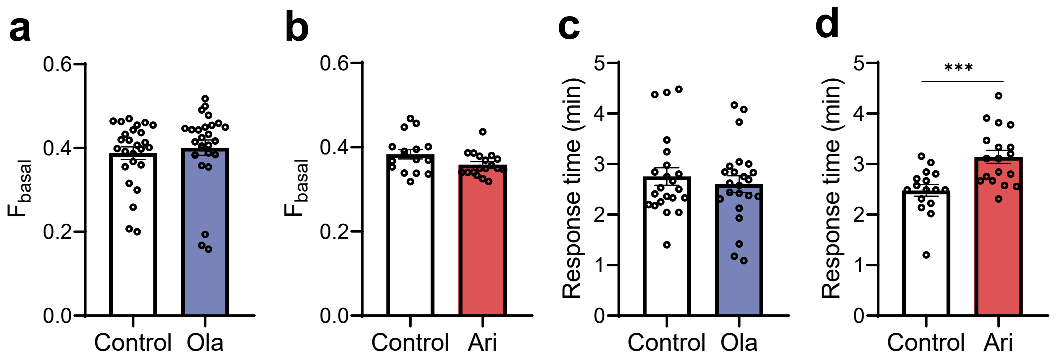

ESM Figure 3. Ex vivo treatment with aripiprazole delays the response time of glucose-induced Ca^{2+} signals in pancreatic islets from female mice. **(a-b)** Basal fluorescence measured in the absence of high glucose stimulation (F_{basal}; measured at 2.8 mmol/l glucose) of control *vs.* islets treated with 6 µmol/l olanzapine **(a)** or aripiprazole **(b)**. **(c-d)** Response time of islets treated with vehicle *vs.* olanzapine **(c)** or aripiprazole **(d)**. Data are presented as mean ± SEM. A total of 16-26 islets were used in each condition (n= 6 mice). P-values were determined by student's *t*-test or Mann-Whitney's test. ***p<0.001 compared to islets exposed to vehicle (0.01% DMSO).

References

[1] Ribas-Aulinas F. Ribo S. Parra-Vargas M. et al. (2021) Neonatal overfeeding during lactation rapidly and permanently misaligns the hepatic circadian rhythm and programmes adult NAFLD. Molecular metabolism 45: 101162. 10.1016/j.molmet.2021.101162
